# Firearm injuries in a conflict affected region: A multicenter study of epidemiological trends, injury patterns, management, and outcomes in the West Bank of Palestine

**DOI:** 10.1371/journal.pone.0358566

**Published:** 2026-09-22

**Authors:** Hala Alkhoury, Mira Hallak, Mohammad Shakshir, Iyad Maqboul, Mohammad Jaber, Ramzi Shawahna

**Affiliations:** 1 Department of Medicine, Faculty of Medicine and Health Sciences, An-Najah National University, Nablus, Palestine; 2 An-Najah National University Hospital, Nablus, Palestine; 3 Department of Physiology, Pharmacology, and Toxicology, Faculty of Medicine and Health Sciences, An-Najah National University, Nablus, Palestine; 4 Clinical Research Center, An-Najah National University Hospital, Nablus, Palestine; University of Alabama at Birmingham, UNITED STATES OF AMERICA

## Abstract

Firearm injuries represent a major source of trauma in conflict‑affected regions, yet multicenter data from Palestine remain scarce. This multicenter study provides the first large epidemiological analysis of firearms injuries across eight hospitals in the West Bank, encompassing 392 patients treated between 2020 and 2025. The cohort was overwhelmingly male (98.0%) and predominantly young, with a median age of 19.0 years [17.0–26.0]. Extremity injuries were most common, affecting 265 (67.6%) patients, while thoracoabdominal and vascular injuries, though less frequent, were associated with disproportionately severe outcomes. Fractures were the most frequent associated injury, affecting 152 patients (38.8%), followed by vascular trauma in 54 patients (13.8%) and hollow viscus gastrointestinal injuries in 45 patients (11.5%). Surgical interventions were required in more than half of patients, with orthopedic fixation and vascular repair dominating the workload. During hospitalization, 81 (20.7%) patients required intensive care unit (ICU) admission, while in‑hospital mortality was 3.3%. Functional dependence at discharge was common, affecting more than half of survivors. Multivariable regression showed that elevated heart rate at presentation was independently associated with ICU admission (OR: 1.04, 95% CI: 1.02–1.05, p < 0.001). Abdominal injuries were also significant predictors of ICU admission (OR: 3.81, 95% CI: 1.64–8.87, p = 0.002). Increasing age (OR: 0.93, 95% CI: 0.87–0.99, p = 0.024) and elevated heart rate (OR: 0.93, 95% CI: 0.90–0.97, p = 0.001) were independently associated with in‑hospital mortality. This study shows that while extremity injuries dominate the epidemiological profile of firearm trauma in the West Bank, thoracoabdominal and vascular injuries were associated with greater resource demands and more severe outcomes. The findings underscore the urgent need to strengthen orthopedic and vascular surgical capacity, expand ICU and blood bank resources, and develop rehabilitation services.

## Introduction

Firearm injuries remain a major cause of preventable death and disability worldwide, exerting a disproportionate impact on trauma systems in regions affected by violence and instability [1,2]. According to a Global Burden of Disease analysis, firearm violence accounted for over 3.1 million incident cases worldwide in 2019, including 710,600 cases of physical violence by firearm, 335,200 cases of firearm-related self-harm, and 2.1 million unintentional firearm injuries [3]. The age‑standardized mortality rate from firearm violence was 2.23 per 100,000 population, with disability-adjusted life years (DALYs) reaching 127.6 per 100,000 for physical violence alone [3]. Importantly, firearm injuries disproportionately affect younger populations, ranking among the leading causes of death in individuals aged 5–29 years, and show increasing trends in regions such as Tropical Latin America and the Middle East/North Africa, where socioeconomic instability and conflict exacerbate risk [3,4].

From a clinical perspective, firearm injuries differ from other mechanisms of trauma because their severity depends on ballistic characteristics, anatomical trajectory, and the timeliness of definitive care [4–6]. Presentations may range from isolated soft‑tissue injuries or fractures to complex vascular, thoracic, abdominal, pelvic, genitourinary, or neurologic involvement [1–4]. This variability necessitates rapid triage, early resuscitation, timely imaging when available, and coordinated multidisciplinary care involving emergency medicine, surgery, orthopedics, vascular teams, critical care, and rehabilitation services [7,8].

Although firearm injuries are a global concern, their burden is particularly pronounced in low‑resource and conflict‑affected regions, where fragile health systems face limited prehospital care, constrained surgical capacity, and inadequate rehabilitation services [3]. The Middle East and Africa have been identified as regions with rising age‑standardized rates of firearm violence, reflecting the combined effects of political instability, socioeconomic disparities, and recurrent episodes of armed conflict [9–11]. In these settings, patients often present directly to hospitals without prior stabilization, and trauma registries are either absent or incomplete, limiting the ability to anticipate resource needs or benchmark outcomes. Within Palestine, and specifically the West Bank, firearm injuries represent a significant form of penetrating trauma, yet multicenter data describing injury patterns, management strategies, and outcomes remain scarce. Addressing this gap is essential for strengthening trauma system preparedness, guiding resource allocation, and informing policy interventions tailored to the realities of a conflict‑affected healthcare environment.

In the Middle East, firearm injuries have been consistently reported as a leading cause of trauma-related morbidity and mortality, particularly in conflict-affected settings where health systems face chronic resource limitations [9,10]. Regional studies highlight the predominance of young male victims, high rates of extremity injuries, and the frequent need for surgical interventions such as vascular repair, laparotomy, and orthopedic fixation. In Palestine, the burden of firearm injuries has been documented in both the West Bank and Gaza, with several reports describing severe thoracoabdominal trauma, vascular injuries, and amputations that contribute to long-term disability [10,12–14]. Analyses from Gaza during periods of armed conflict emphasize the overwhelming demand for surgical and critical care services, while smaller case series from the West Bank have noted the disproportionate impact on adolescents and young adults, limited prehospital stabilization, and the absence of standardized trauma registries [10,12–14]. Collectively, these studies underscore the dual challenge of high injury incidence and constrained trauma system capacity, yet most available data remain single-center, event-specific, or descriptive in nature. This lack of multicenter, systematically collected evidence limits the ability to anticipate resource requirements, benchmark outcomes, and design targeted interventions.

Although firearm injuries have been widely recognized as a major contributor to trauma‑related morbidity and mortality in the Middle East [10,12,13], the existing literature from Palestine remains scarce and fragmented despite the persistently high levels of violence. Most available reports have been limited to single‑center case series or conflict‑specific accounts, often focusing narrowly on acute presentations without systematically describing epidemiological trends, injury distribution, management strategies, or hospital outcomes [9,10,12–14]. Critical domains such as intensive care unit (ICU) utilization, transfusion requirements, functional status at discharge, and mortality have rarely been examined in a comprehensive manner, and the absence of standardized trauma registries has further hindered the ability to generate reliable multicenter evidence. In addition, triage of firearm‑related trauma in Palestine remains challenging due to the lack of standardized severity scoring systems, limited prehospital stabilization, and variability in hospital documentation practices. These gaps make it difficult to identify patients at highest risk of requiring intensive care or experiencing in‑hospital mortality, underscoring the need for evidence‑based predictors to guide triage decisions. This gap in knowledge is particularly striking given the recurrent exposure of Palestinian communities to firearm‑related trauma and the strain it imposes on already fragile healthcare systems. To address these limitations, the present study was undertaken as the first multicenter retrospective cohort analysis of firearm injuries in the West Bank, with the objective of providing a detailed account of epidemiological characteristics, injury patterns, management approaches, and in‑hospital outcomes. By consolidating data across eight hospitals, this study aims to generate robust evidence that can inform trauma system preparedness, guide resource allocation, and support policy development in conflict‑affected healthcare settings.

## Methods

### Study design and settings

This study was designed as a multicenter retrospective observational cohort study conducted across hospitals in the West Bank of Palestine. Patients presenting with documented firearm injuries between 2020 and 2025 were included. Data were collected retrospectively from electronic medical records (EMRs), with extraction performed between 01/09/2025 and 30/01/2026 using a standardized data collection form to ensure consistency across centers. The participating hospitals represented a mix of governmental and private institutions geographically distributed across the northern and southern regions of the West Bank. Cases were managed at Rafidia Surgical Hospital, Nablus Specialty Hospital, An-Najah National University Hospital, Beit Jala Governmental Hospital (Al-Hussein), Jenin Governmental Hospital, Tubas Turkish Governmental Hospital, Martyr Dr. Thabet Thabet Governmental Hospital, and the Palestinian Medical Complex. Reporting of this study adhered to the Strengthening the Reporting of Observational Studies in Epidemiology (STROBE) checklist [15], provided as S1 Table, to ensure methodological transparency and alignment with international reporting standards.

### Study population and eligibility criteria

The study population comprised patients with documented firearm injuries who presented to and were managed at the participating hospitals in the West Bank during the study period. Eligible cases required clear documentation of a firearm injury and a verifiable hospital encounter within one of the included centers. Records were excluded if injuries were caused by other mechanisms (e.g., stab wounds, blunt trauma), if patients were treated entirely outside the study hospitals, or if documentation was insufficient to confirm a firearm injury event.

Partial missingness in selected clinical variables (e.g., Glasgow Coma Scale [GCS]) did not result in exclusion. Instead, missing data were quantified and transparently reported, with analyses conducted using available-case denominators for each variable. Patients were followed from hospital admission until discharge, transfer, or death during the index hospitalization.

### Sample size and sampling approach

The sample size required for this study was estimated using the Raosoft sample size calculator (https://www.raosoft.com), assuming a 95% confidence level, a 5% margin of error, and a population size approximating all patients presenting with firearm injuries to hospitals in the West Bank during the study period. Based on these parameters, the minimum required sample size was calculated to be 385 participants, ensuring sufficient statistical power to generate stable descriptive estimates and allow meaningful subgroup analyses.

To maximize representativeness, all eligible cases documented between 2020 and 2025 across the eight participating hospitals were included. No sampling or case selection was performed beyond the predefined eligibility criteria, thereby minimizing selection bias. The final analytic dataset comprised 392 patients with confirmed firearm injuries, exceeding the calculated minimum requirement. This complete inclusion approach strengthened the external validity of the findings and ensured that rare injury types and less frequent outcomes were adequately captured.

### Definition of variables

Variables were defined *a priori* based on clinical relevance and consistency with published firearm trauma literature. Baseline demographic variables included sex, age, and place of residence. Temporal variables captured the year, month, and day of presentation. Prehospital medical attention was also collected. Prehospital medical attention was categorized into three groups: “none” (patients arriving without any documented care prior to hospital presentation), “first aid” (basic wound care or stabilization provided at the scene or by non‑specialized personnel), and “care in another hospital/center” (patients who received initial medical or surgical management in a different facility before transfer to the study hospital).

Clinical status at emergency presentation included blood pressure, heart rate, hemodynamic stability, and neurological status. Blood pressure was categorized pragmatically to ensure consistency across hospitals. We applied American College of Cardiology (ACC)/American Heart Association (AHA) thresholds, adapted to trauma settings, as follows: hypotension (systolic blood pressure <90 mmHg), normotension (systolic blood pressure 90–139 mmHg with diastolic blood pressure <90 mmHg), and hypertension (systolic blood pressure ≥140 mmHg or diastolic blood pressure ≥90 mmHg) [16]. These categories were used to provide a standardized and reproducible framework for analysis rather than to imply chronic cardiovascular risk. In the context of firearm trauma, this classification was intended to capture acute physiological status at presentation, recognizing that trauma‑specific thresholds (such as systolic blood pressure <90 mmHg for shock) may be more clinically meaningful. Heart rate was categorized as bradycardia (< 60 bpm), normal (60–100 bpm), or tachycardia (> 100 bpm). Hemodynamic instability was defined as heart rate <50 or > 120 bpm, systolic blood pressure <90 mmHg, or diastolic blood pressure <60 mmHg. Neurological status was assessed separately using the Glasgow Coma Scale [17], categorized as ≥13, 9–12, 3–8, or not recorded.

Injury variables were classified by anatomical region and by injury type. Associated injuries and fractures were also classified by anatomical region and by injury type. Management variables included wound management, bullet/foreign body removal, fracture stabilization/fixation, and surgical interventions were also collected.

Outcome variables included ICU admission, blood transfusion, antibiotic administration, length of hospital stay, discharge disposition, functional status at discharge, and mortality status (alive/deceased). Functional status at discharge was assessed based on documentation in medical records and categorized into three groups: “independent” (patients discharged with preserved mobility and self‑care capacity without the need for assistance), “dependent” (patients discharged with impaired mobility, need for caregiver support, or functional limitations requiring rehabilitation services), and “deceased.” This pragmatic categorization reflects routine clinical documentation practices in the participating hospitals and was used to capture functional outcomes in the absence of standardized assessment instruments.

### Data collection

Data were abstracted using a standardized, customized collection form developed specifically for this study (S2 Table). The form captured demographics, injury characteristics, physiological parameters, comorbidities, interventions, and outcomes. A structured variable dictionary accompanied the form, defining each variable and its coding framework to ensure reproducibility.

Data abstraction was performed retrospectively from EMRs by trained research team members who were final year medical students. Abstractors underwent orientation to the standardized protocol and variable dictionary to ensure consistent interpretation across hospitals. Systematic data cleaning procedures were applied to harmonize category labels, standardize anatomical and procedural terminology, and perform logical consistency checks (e.g., alignment between recorded procedures and number of surgeries).

Missing data were preserved as a distinct category (not recorded) rather than reclassified, minimizing misclassification bias and maintaining transparency. The approach was informed by methodologies commonly used in retrospective trauma research and relied on routinely documented clinical records generated during standard patient care.

### Statistical analysis

Data were coded, cleaned, and analyzed using IBM SPSS Statistics for Windows, version 26. Categorical variables were summarized as frequencies (n) and percentages (%), while continuous variables were reported as median with interquartile range [Q1, Q3].

Bivariate analyses were conducted to explore associations between baseline characteristics, injury patterns, management procedures, and clinical outcomes. For categorical comparisons, Pearson’s chi-square test was applied, with Fisher’s exact test used when expected cell counts were <5. For continuous outcomes compared between two groups, Mann-Whitney U tests were used. For comparisons across more than two groups, Kruskal-Wallis tests were employed.

To identify factors independently associated with ICU admission and in‑hospital mortality, multivariable logistic regression models were constructed. Candidate variables were selected a priori through a two‑step process: (1) screening for statistical significance in bivariate analyses (p < 0.10), and (2) evaluation of clinical relevance based on prior trauma literature. Specifically, age and pulse rate on arrival were included because they are consistently reported as strong predictors of mortality and ICU utilization [2,6,9,10]. Chest trauma and abdominal trauma were entered separately into the models, as both regions are recognized in global and regional firearm injury studies as major determinants of severity and resource demand [2,6,9,10,12–14]. Other variables (e.g., limb injuries, soft tissue injuries, minor fractures) were excluded because they did not reach statistical significance in univariate analyses, lacked established evidence of independent prognostic value, or were collinear with included predictors. Given the small number of deaths (n = 13), the mortality model was deliberately restricted to a parsimonious set of variables to minimize overfitting. This strategy ensured that the models balanced explanatory power with interpretability while maintaining transparency.

Model fit was assessed using the Hosmer-Lemeshow goodness-of-fit test, and explanatory power was reported using Cox & Snell R^2^ and Nagelkerke R^2^. Odds ratios (ORs) with 95% confidence intervals (CIs) were calculated to quantify associations. To further evaluate model performance, the area under the curve (AUC) was reported to assess discriminatory ability.

Missing data were not imputed; instead, analyses were conducted using available-case denominators, allowing denominators to vary by variable. Missing or non-recordable fields were explicitly reported in tables and supplementary materials to maintain transparency. All statistical tests were two-sided, and a p-value < 0.05 was considered statistically significant.

### Ethical considerations

This study was conducted in full compliance with national and international ethical principles governing biomedical research. All procedures adhered to the ethical standards outlined in the Declaration of Helsinki. Formal approval was obtained from the Institutional Review Board of An‑Najah National University (approval number: Pharm. Dec.2024/60). In addition, administrative permissions were secured from all participating hospitals prior to data collection.

Because this study involved a retrospective analysis of routinely collected clinical data, informed consent requirements were addressed in accordance with IRB regulations. In view of the observational and non‑interventional design, and the exclusive reliance on existing medical records, the Institutional Review Board of An‑Najah National University formally waived the requirement for obtaining written informed consent. All data were managed in strict accordance with confidentiality and data protection standards. Patient identifiers were removed prior to analysis, and datasets were fully de‑identified and stored on secure, access‑restricted devices available only to the research team. These measures ensured that data were used exclusively for research purposes and safeguarded patient privacy throughout the study.

## Results

### Characteristics of the patients in the cohort

The cohort included 392 patients with firearm injuries, of whom the vast majority were male (n = 384; 98.0%) (Table 1). The median age was 19.0 years [17.0, 26.0], reflecting a predominantly young population. More than half of the cohort were young adults aged 18–29 years (n = 201; 51.3%), while adolescents aged 13–17 years represented nearly one‑third of the population (n = 122; 31.1%). Pediatric patients aged ≤12 years were less common (n = 10; 2.6%), and older adults aged ≥45 years accounted for only a small minority (n = 16; 4.1%). These findings underscore that while firearm injuries affect individuals across age groups, the largest burden was observed among young adults aged 18–29 years, who comprised more than half of the cohort. The full distribution across age groups is presented in S1 Fig.

**Table 1:** Demographic characteristics and clinical presentation of the study cohort (*n = 392*).

Variable	n (%) or median [Q1, Q3]
Gender	
Male, n (%)	384 (98.0)
Female, n (%)	8 (2.0)
Age (years), median [Q1, Q3]	19.0 [17.0-26.0]
Place of Residence	
City, n (%)	175 (44.6)
Village, n (%)	96 (24.5)
Refugee Camp, n (%)	78 (19.9)
Undisclosed/not documented, n (%)	43 (11.0)
Blood pressure	
Non-recordable/not documented, n (%)	9 (2.3)
Normotensive, n (%)	176 (44.9)
Hypotensive, n (%)	38 (9.7)
Hypertensive, n (%)	169 (43.1)
Heart rate	
Non-recordable/not documented, n (%)	7 (1.8)
Normal, n (%)	292 (74.5)
Bradycardia, n (%)	4 (1.0)
Tachycardia, n (%)	89 (22.7)
Glasgow coma scale recorded at time of arrival to the emergency room	
> 13, n (%)	308 (78.6)
9–12, n (%)	19 (4.8)
3–8, n (%)	13 (3.3)
Not recorded/not documented, n (%)	52 (13.3)
Hemodynamic stability of the patient at the time of arrival to the emergency room	
Stable, n (%)	314 (80.1)
Unstable, n (%)	78 (19.9)
Medical attention patient received prior to arriving to the emergency room	
None/not documented, n (%)	46 (11.7)
First aid, n (%)	305 (77.8)
Care in another hospital/center, n (%)	41 (10.5)

Q1: first quartile, Q3: third quartile.

Nearly half of the cohort resided in cities (n = 175; 44.6%), while villages (n = 96; 24.5%) and refugee camps (n = 78; 19.9%) (Table 1). At presentation, most patients were hemodynamically stable (n = 314; 80.1%), although instability was documented in 78 (19.9%) patients. Normotension was the most frequent blood pressure category (n = 176; 44.9%), followed closely by hypertension (n = 169; 43.1%). Tachycardia was observed in 89 (22.7%) patients. Neurological status was generally preserved, with GCS scores > 13 in 308 (78.6%) patients, while moderate in 19 patients (4.8%) and severe impairment in 13 (3.3%). Pre‑arrival care was documented in most cases, with 305 (77.8%) receiving first aid, whereas 46 (11.7%) arrived without prior medical attention.

### Temporal distribution of firearm injury presentations

**Fig 1:**
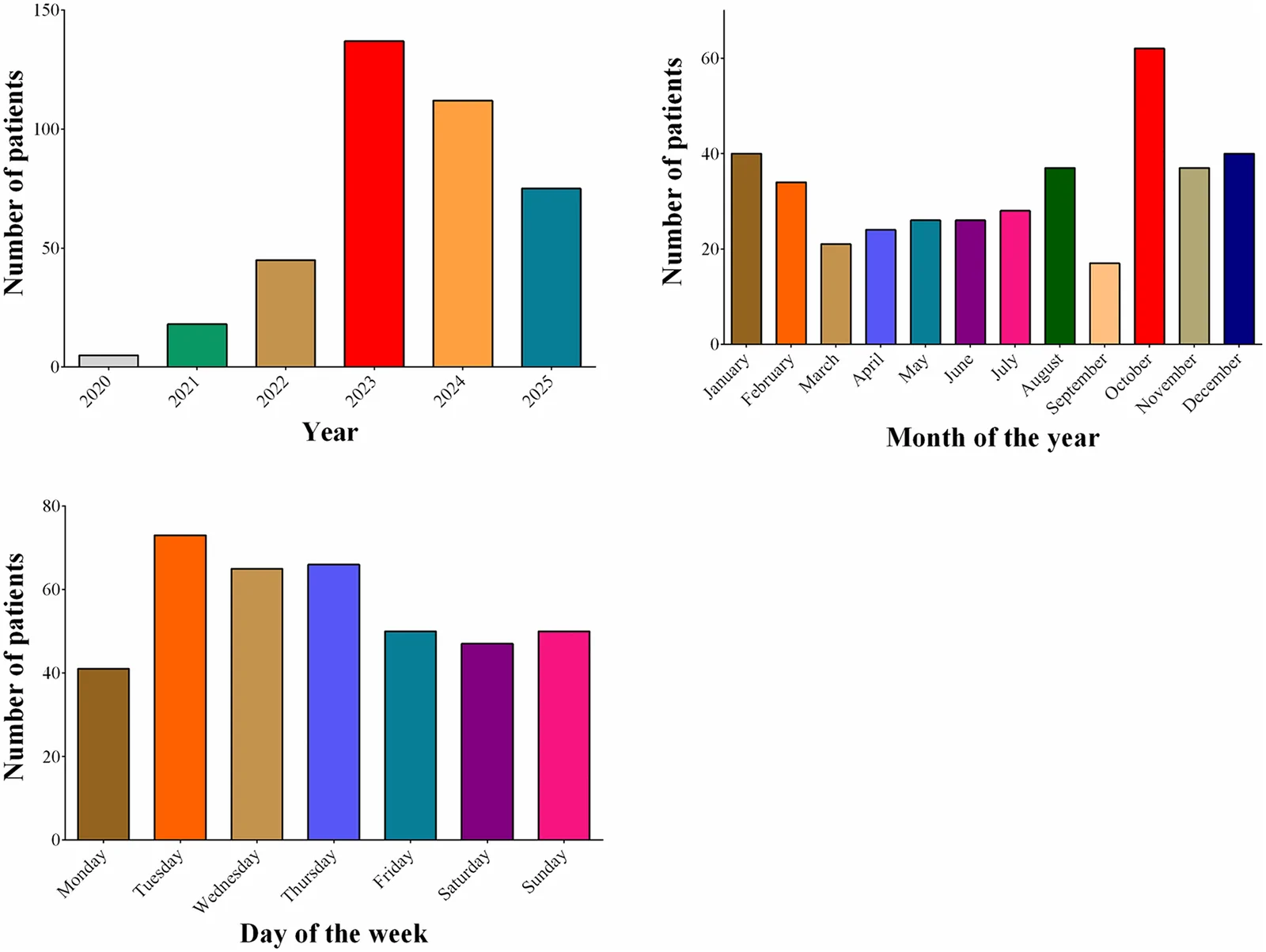
Temporal distribution of firearm injury presentations by year, month, and day of the week.

The occurrence of firearm injury presentations varied considerably across years, months, and days of the week (Fig 1). The highest burden was observed in 2023, with 137 (34.9%) cases, followed by 2024 with 112 (28.6%) and 2025 with 75 (19.1%). Earlier years contributed fewer cases, including 2022 (n = 45; 11.5%), 2021 (n = 18; 4.6%), and 2020 (n = 5; 1.3%). Monthly distribution revealed a peak in October (n = 62; 15.8%), while March (n = 21; 5.4%) and September (n = 17; 4.3%) recorded the lowest frequencies. Weekly distribution showed that Tuesday accounted for the largest proportion of cases (n = 73; 18.6%), whereas Monday had the fewest (n = 41; 10.5%). These findings highlight temporal clustering, with notable surges in specific years, months, and weekdays, while the full distribution is detailed in Fig 1.

### Anatomical distribution of injuries

**Fig 2:**
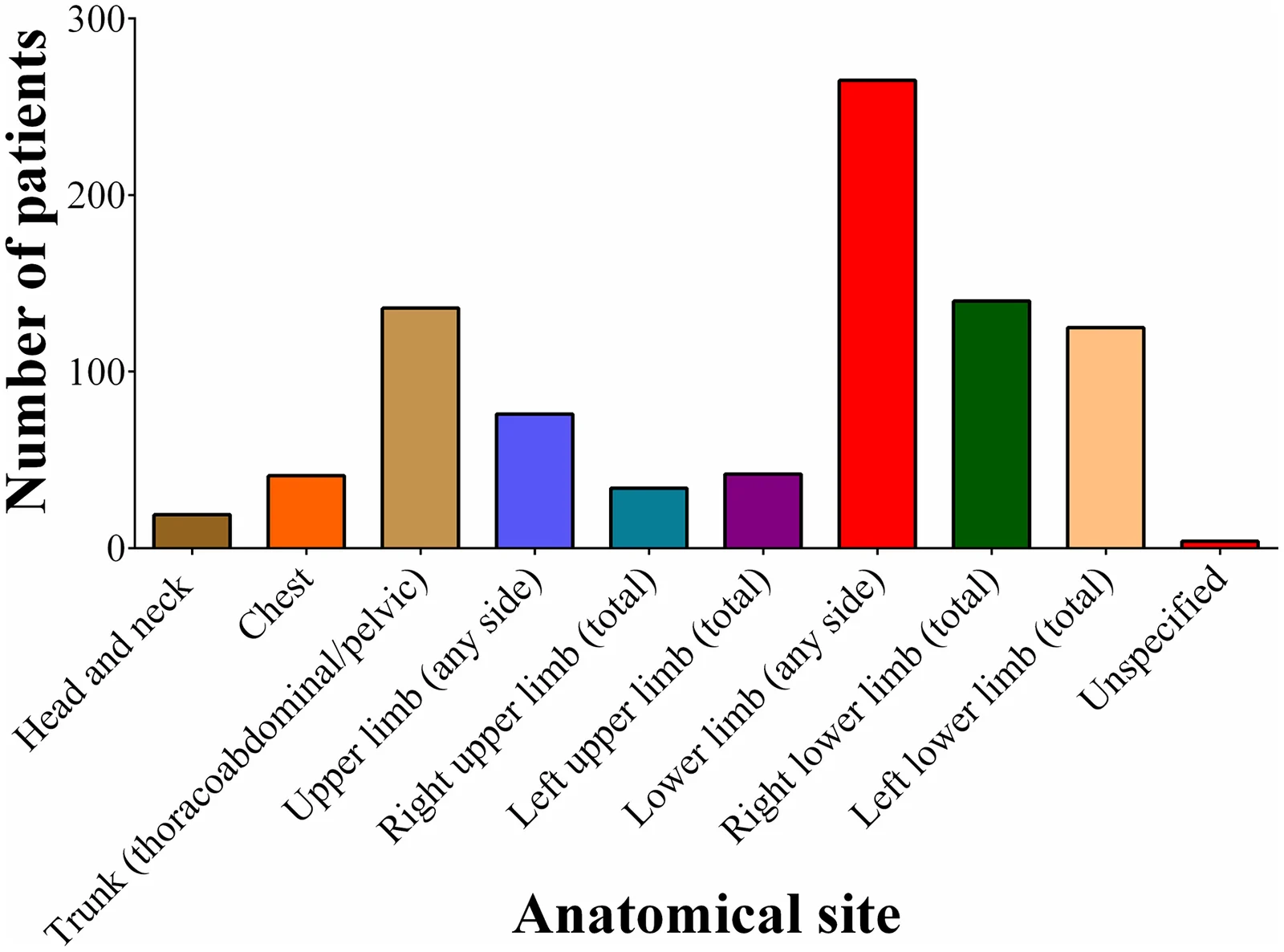
Distribution of firearm injury by anatomical location and body region. Anatomical injury locations are not mutually exclusive, and individual patients may have sustained injuries in more than one anatomical region.

Firearm injuries most frequently involved the lower extremities, affecting 265 (67.4%) patients, with nearly equal distribution between the right (n = 140; 35.6%) and left (n = 125; 31.8%) sides (Fig 2). Injuries to the trunk, including thoracoabdominal and pelvic regions, were also common (n = 136; 34.6%), while upper limb injuries were documented in 76 (19.3%). Within the upper limb category, left‑sided injuries (n = 42; 10.7%) slightly outnumbered right‑sided injuries (n = 34; 8.7%). Less frequent sites included the chest (n = 41; 10.5%) and head/neck (n = 19; 4.8%). Only a small proportion of cases were classified as unspecified (n = 4; 1.0%). These findings highlight the predominance of extremity involvement, particularly the lower limbs, with trunk injuries representing the second most common anatomical region (Fig 2). A detailed breakdown of injury distribution by anatomical site is shown in S3 Table.

### Associated injuries

Associated injuries were common among patients with firearm injuries, with fractures representing the most frequent category (n = 152; 38.8%) (Table 2). Within fracture subtypes, tibial fractures (n = 38; 9.7%) and femoral fractures (n = 32; 8.2%) were the most prevalent, followed by upper limb fractures (n = 21; 5.4%) and pelvic fractures (n = 17; 4.3%). Vascular injuries were also notable, affecting 54 (13.8%) patients, while hollow viscus gastrointestinal injuries were documented in 45 (11.5%). Thoracic injuries occurred in 39 (10.0%), and abdominal solid organ injuries were observed in 25 (6.4%). Less frequent but clinically significant injuries included nerve injury (n = 15; 3.8%), genitourinary injury (n = 17; 4.3%), vertebral column fracture (n = 8; 2.0%), and cranial or intracranial injury (n = 9; 2.3%). Rare injuries such as ruptured globe (n = 2; 0.5%) and major abdominal vascular injury (n = 3; 0.8%) were also recorded. These findings highlight the predominance of fractures and vascular involvement, with thoracoabdominal injuries contributing substantially to morbidity. A detailed breakdown of associated injuries is shown in S4 Table.

**Table 2:** Distribution of injuries among the study cohort.

Associated injury	n (%)^*^
Vascular injury	54 (13.8)
Nerve injury	15 (3.8)
Thoracic injury (any)	39 (10.0)
Cranial and intracranial injury (any)	9 (2.3)
Facial bone fracture (any)	7 (1.8)
Ruptured globe	2 (0.5)
Neck injury (any)	6 (1.5)
Vertebral column fracture (any)	8 (2.0)
Thoracic girdle fracture (any)	12 (3.1)
Diaphragmatic injury	6 (1.5)
Abdominal solid organ injury (any)	25 (6.4)
Hollow viscus gastrointestinal injury (any)	45 (11.5)
Major abdominal vascular injury (any)	3 (0.8)
Pelvic/retroperitoneal hematoma (any)	8 (2.0)
Genitourinary injury (any)	17 (4.3)
All fractures (any location)	152 (38.8)
Pelvic fracture (any)	17 (4.3)
Femoral fracture (any)	32 (8.2)
Tibial fracture (any)	38 (9.7)
Foot and ankle fracture (any)	11 (2.8)
Upper limb fracture (any)	21 (5.4)
Hand injury (any)	2 (0.5)
Tendon injury (any)	4 (1.0)
Muscle injury	9 (2.3)

* Percentages were calculated using the total number of patients in the cohort (*n = 392*). Injury categories are not mutually exclusive, and individual patients may have sustained multiple injuries.

### Management and discharge outcomes

Surgical procedures were frequently required in the management of firearm injuries, with fracture stabilization or fixation being the most common intervention (n = 92; 23.5%) (Table 3). Vascular surgery was also prominent, performed in 64 (16.3%), while wound management procedures were documented in 64 (16.3%). Exploratory surgeries were undertaken in 60 (15.3%), and abdominal visceral surgeries in 44 (11.2%). Bullet or foreign body removal was performed in 44 (11.2%), highlighting the complexity of trauma care. Less frequent but clinically significant procedures included thoracic surgery (n = 17; 4.3%), genitourinary/scrotal surgery (n = 11; 2.8%), tendon/nerve repair (n = 13; 3.3%), and neurosurgical interventions (n = 5; 1.3%). Rare procedures such as tracheostomy (n = 1; 0.3%) and ocular evisceration (n = 1; 0.3%) were also recorded. These findings underscore the predominance of orthopedic and vascular interventions, reflecting the injury burden in this cohort. A detailed breakdown of surgical and management interventions is shown in S5 Table.

**Table 3:** Surgical interventions performed in patients in the cohort.

Surgical intervention	n (%)^*^
Wound management (any)	64 (16.3)
Bullet or foreign body removal	44 (11.2)
Grafting procedures (any)	6 (1.5)
Fracture stabilization/fixation (any)	92 (23.5)
Exploratory surgery (any)	60 (15.3)
Thoracic surgery (any)	17 (4.3)
Vascular surgery (any)	64 (16.3)
Endoscopic procedures (any)	4 (1.0)
Fasciotomy	8 (2.0)
Neurosurgical intervention (any)	5 (1.3)
Tracheostomy	1 (0.3)
Ocular evisceration	1 (0.3)
Abdominal visceral surgery (any)	44 (11.2)
Genitourinary/scrotal surgery (any)	11 (2.8)
Tendon/nerve surgery (any)	13 (3.3)
Maxillofacial surgery	2 (0.5)
Other surgical interventions	9 (2.3)

* Percentages were calculated using the total number of patients in the cohort (*n = 392*). Surgical procedure categories are not mutually exclusive, and individual patients may have undergone more than one procedure during admission.

During hospitalization, 81 (20.7%) patients required admission to the ICU, and 106 (27.0%) received blood products (Table 4). Antibiotic therapy was administered to nearly all patients (n = 367; 93.6%). Surgical intervention was common, with 228 (58.2%) undergoing at least one procedure; specifically, 122 (31.1%) had a single surgery, 75 (19.1%) had two, and 31 (7.9%) required three or more. At discharge, most patients returned home (n = 304; 77.6%), while 59 (15.1%) were referred to other facilities. In‑hospital mortality was documented in 13 (3.3%), and 16 (4.1%) left against medical advice. Functional outcomes revealed that only 177 (45.2%) were discharged as independent with normal status, whereas more than half (n = 202; 51.5%) were dependent, underscoring the substantial morbidity associated with firearm trauma.

**Table 4:** Clinical management and discharge outcomes of patients in the study cohort.

Variable	n (%) or median [Q1, Q3]
ICU admission, n (%)	81 (20.7)
Blood transfusion, n (%)	106 (27.0)
Antibiotic administration, n (%)	367 (93.6)
Number of surgeries	
None, n (%)	164 (41.8)
1, n (%)	122 (31.1)
2, n (%)	75 (19.1)
≥ 3, n (%)	31 (7.9)
Discharge status	
Home, n (%)	304 (77.6)
Referral, n (%)	59 (15.1)
Died, n (%)	13 (3.3)
Left against medical advice, n (%)	16 (4.1)
Length of hospital stay (days), median [Q1, Q3]	3.0 [1.0, 6.0]
Functional outcome at discharge	
Independent, n (%)	177 (45.2)
Dependent, n (%)	202 (51.5)
Dead, n (%)	13 (3.3)

ICU, intensive care unit.

### Factors associated with ICU admission and mortality

In univariate analyses, patients admitted to the ICU had significantly higher heart rates on arrival (median 100.0 [87.0–122.0] vs. 87.0 [78.0–93.0] beats/min, p < 0.001), and both chest injuries (p < 0.001) and abdominal injuries (p < 0.001) were more frequent among patients admitted to the ICU as shown in S6 Table. In multivariate analysis, heart rate on arrival remained independently associated (Table 5), with each unit increase associated with higher odds of ICU admission (B = 0.04, p < 0.001; OR = 1.04, 95% CI: 1.02–1.05). Abdominal injuries were also independently associated with ICU admission (B = 1.34, p = 0.002; OR = 3.81, 95% CI: 1.64–8.87). The overall model was statistically significant (χ^2^ = 58.35, df = 3, p < 0.001), demonstrated acceptable calibration (Hosmer–Lemeshow χ^2^ = 11.79, p = 0.161), and yielded an AUC of 0.78, indicating good discriminatory ability. The model correctly classified 81.3% of cases, underscoring the importance of hemodynamic parameters and torso trauma in determining the need for intensive care.

**Table 5:** Factors independently associated with ICU admission and mortality.

					95% CI for OR	
**Domain/factor**	**B**	**SE**	**p-value**	**OR**	**Lower**	**Upper**
**ICU admission**						
Heart rate on arrival	0.04	0.01	**< 0.001**	1.04	1.02	1.05
Chest injury	−0.66	0.47	0.163	0.52	0.21	1.30
Abdomen (any)	1.34	0.43	**0.002**	3.81	1.64	8.87
**Mortality**						
Age	−0.07	0.03	**0.024**	0.93	0.87	0.99
Heart rate on arrival	−0.07	0.02	**0.001**	0.93	0.90	0.97

CI, confidence interval; ICU, intensive care unit; OR, odds ratio; SE, standard error; statistically significant *p*-values are in boldface.

Similarly, univariate analyses showed that patients who died were significantly older (median 30.5 [29.0–35.0] years vs. 19.0 [17.0–26.0] years, p = 0.004) and had markedly higher heart rates on arrival (median 113.5 [112.0–130.0] vs. 87.0 [79.0–99.0] beats/min, p = 0.002) (S7 Table). In multivariate analysis, increasing age was associated with higher likelihood of death (B = –0.07, p = 0.024; OR = 0.93, 95% CI: 0.87–0.99), and elevated heart rate on arrival was independently associated with mortality (B = –0.07, p = 0.001; OR = 0.93, 95% CI: 0.90–0.97) (Table 5). The overall model was statistically significant (χ^2^ = 14.84, df = 2, p = 0.001), demonstrated acceptable fit (Hosmer–Lemeshow χ^2^ = 2.99, p = 0.93), and yielded an AUC of 0.81, indicating good discriminatory ability. The model correctly classified 98.4% of cases.

## Discussion

This study provides the first multicenter epidemiological analysis of firearm injuries in the West Bank of Palestine, offering a comprehensive account of injury patterns, management strategies, and outcomes across eight hospitals. By analyzing 392 patients treated between 2020 and 2025, it delivers unprecedented insight into the burden of firearm trauma in a conflict‑affected healthcare system. The findings highlight the predominance of extremity injuries, the disproportionate severity of thoracoabdominal and vascular trauma, the high demand for surgical and critical care interventions, and the substantial functional dependence among survivors despite relatively low in‑hospital mortality. These results are directly relevant to clinicians, hospital administrators, trauma system planners, and policymakers, underscoring the urgent need to strengthen surgical capacity, expand intensive care resources, and develop rehabilitation services. Beyond their local importance, the findings contribute to the global discourse on trauma care in conflict zones, offering evidence to guide preparedness strategies and policy development in similarly constrained environments.

The predominance of extremity injuries observed in this cohort is consistent with international firearm injury series, where limbs are often the most exposed and vulnerable anatomical regions [1,5]. Regional studies report similar extremity‑dominant profiles, while reports from Gaza have documented higher proportions of thoracoabdominal trauma and vascular involvement [10,12–14]. Although less frequent in our cohort, thoracoabdominal and vascular injuries were associated with disproportionately severe outcomes in descriptive analyses, including higher rates of ICU admission, transfusion, and mortality. In multivariable models, abdominal injuries remained significantly associated with ICU admission. These associations, together with the observed burden of vascular trauma, highlight the need to strengthen orthopedic and vascular surgical capacity, ensure adequate critical care resources, and expand rehabilitation services to address long‑term disability [2,6].

Associated injuries, particularly fractures and vascular trauma, highlight the substantial burden on surgical services. Extremity fractures, especially tibial and femoral, were the most frequent injuries, consistent with both civilian firearm trauma series and regional reports from Gaza [12,13]. Vascular injuries, though less common, carried significant clinical implications, echoing studies that underscore the urgent need for repair and transfusion support [10,14]. Thoracoabdominal injuries, while less frequent, were associated with severe outcomes in descriptive analyses, consistent with global trauma literature emphasizing the high mortality risk of torso involvement [2,6]. Taken together, these findings suggest that orthopedic and vascular surgical services are critical components of trauma care in this setting. The observed associations with ICU admission and transfusion further indicate that critical care and blood bank resources are frequently required. Establishing multicenter trauma registries would enable systematic monitoring of associated injuries, facilitate benchmarking against regional and international data, and guide evidence‑based resource allocation [9,10].

Management strategies and discharge outcomes reveal both strengths and vulnerabilities within the trauma care system. The high frequency of orthopedic and vascular procedures parallels findings from regional studies in Gaza, where extremity fractures and vascular trauma dominate the surgical workload [12,13]. International reports similarly emphasize orthopedic fixation and vascular repair as central to firearm trauma care [1,5]. Despite relatively low in‑hospital mortality (3.3%), more than half of survivors were discharged with functional dependence, consistent with regional literature documenting long‑term disability in resource‑limited settings [6,9]. In this cohort, the combination of complex surgical needs, intensive care utilization, transfusion requirements, and high rates of functional dependence underscores the importance of structured rehabilitation programs. Strengthening rehabilitation services, alongside surgical and critical care infrastructure, is therefore a justified priority to improve long‑term outcomes for survivors of firearm trauma.

Associations with ICU admission and mortality provide important insights into early risk stratification. Elevated heart rate at presentation was consistently associated with both ICU admission and mortality, in line with global trauma literature identifying tachycardia and hypotension as markers of physiological compromise [2,6]. Abdominal injuries showed significant associations with ICU admission, reflecting the severity of intra‑abdominal firearm trauma and its need for emergent laparotomy and intensive postoperative monitoring. In contrast, chest injuries did not reach statistical significance in multivariable analysis, though descriptive findings suggest they remain clinically important. Comparable studies have reported that abdominal and thoracic injuries are associated with higher transfusion requirements, prolonged ICU stays, and increased mortality [1,5,12,13]. These exploratory findings highlight the importance of structured triage protocols that integrate hemodynamic assessment and anatomical injury site to identify patients at higher risk. Hospitals in Palestine should prioritize rapid stabilization and expedited surgical intervention for patients presenting with tachycardia or abdominal trauma, while ensuring adequate ICU capacity. At the system level, multicenter trauma registries would allow continuous monitoring of associations and outcomes, enabling refinement of triage algorithms and benchmarking against regional and international standards [9,10].

In summary, this study differs from previous reports by providing the first multicenter, systematically collected evidence on firearm injuries in the West Bank. Unlike earlier single‑center or conflict‑specific case series, it offers consolidated data across eight hospitals, standardized variable definitions, and transparent statistical analyses. By documenting epidemiological trends, injury patterns, management strategies, and outcomes, it fills a critical gap in the literature and establishes a foundation for trauma system preparedness, resource allocation, and policy development in Palestine. The observed associations contribute to local understanding and highlight areas for strengthening trauma care, while also offering insights relevant to other conflict‑affected healthcare systems. These findings should be interpreted cautiously, particularly regarding exploratory regression analyses, but they nonetheless provide valuable lessons that can inform both regional and global approaches to firearm trauma.

### Strengths and limitations

This study has several notable strengths. First, it represents the largest multicenter cohort of firearm injuries ever reported from the West Bank, encompassing eight hospitals and nearly 392 patients, which enhances the generalizability of the findings. Second, the use of a standardized data collection form and variable dictionary ensured consistency across centers, reducing misclassification and improving methodological rigor. Third, the inclusion of both governmental and private hospitals provided a diverse representation of trauma care settings, capturing variability in resources and practices. Fourth, the study adhered to the STROBE reporting guidelines, strengthening transparency and alignment with international standards. Fifth, the analysis incorporated detailed outcome measures, including ICU admission, transfusion, functional status, and mortality, providing a comprehensive picture of the clinical burden beyond simple descriptive accounts. Finally, by situating the findings within regional and global literature, the study offers comparative insights that are directly relevant to trauma system planning in conflict‑affected environments.

Nonetheless, several limitations must be acknowledged. First, the retrospective design relied on medical record documentation, which may have introduced information bias due to incomplete or inconsistent recording of clinical variables. Second, the absence of a national trauma registry limited the ability to capture cases treated outside the participating hospitals, potentially underestimating the true burden. Third, blood pressure was categorized using ACC/AHA thresholds, which are designed for chronic cardiovascular risk stratification and may not be clinically meaningful in the context of acute firearm trauma. This pragmatic choice was made to ensure consistency across hospitals, but future studies should incorporate trauma‑specific measures such as systolic blood pressure thresholds for shock, the Shock Index, or standardized injury severity scores to better capture acute physiological derangements. Fourth, the study did not include long‑term follow‑up, restricting assessment of functional recovery and disability beyond hospital discharge. Fifth, the lack of detailed data on weapon type, ballistic characteristics, and prehospital care constrained interpretation of injury mechanisms and contextual factors. Sixth, while the multicenter design improved representativeness, variability in documentation practices across hospitals may have introduced heterogeneity in data quality. Seventh, functional status was assessed pragmatically from medical record documentation rather than using a standardized instrument such as the Barthel Index or Functional Independence Measure. This may limit comparability with other studies. Future research should incorporate validated functional assessment tools to provide more precise and generalizable measures of disability. Eighth, the study did not include standardized injury severity measures such as the Injury Severity Score, the Abbreviated Injury Scale (AIS), the Revised Trauma Score (RTS), or the Trauma and Injury Severity Score (TRISS). The absence of these widely used scoring systems may have affected the precision of risk stratification and the robustness of multivariable analyses, since residual confounding by injury severity is likely. In addition, the lack of standardized severity adjustment limits comparability with other trauma cohorts and registries, making it difficult to benchmark outcomes across settings. Without these measures, the discriminatory ability and calibration of the prediction models may be influenced by unmeasured injury complexity, and the associations observed should therefore be interpreted with caution. Future studies should incorporate standardized severity scoring systems to improve comparability, strengthen predictive modeling, and reduce residual confounding. Establishing a national trauma registry that systematically collects ISS, AIS, RTS, and TRISS would further enhance external validity and allow more reliable benchmarking of outcomes in conflict‑affected regions. Ninth, the vast majority of injuries were conflict‑related; however, to avoid political polarization and maintain a strictly scientific focus, we did not categorize cases by intent and mechanism of firearm injury (such as conflict‑related, assault, accidental, or self‑inflicted). This limits interpretation of the epidemiological findings, and future studies should incorporate standardized documentation of injury intent and mechanism to enable more nuanced analyses and comparisons across settings. Tenth, the small number of deaths (n = 13) limited the stability of the mortality regression model, raising the potential for model instability and overfitting. Therefore, the mortality findings should be interpreted cautiously and considered exploratory. Future studies should aim to include larger cohorts with higher event counts to enable more robust and stable modeling of mortality outcomes. Establishing a national trauma registry would facilitate comprehensive data collection across hospitals and regions, improving representativeness and statistical power. In addition, prospective multicenter studies with standardized documentation practices and long‑term follow‑up would allow for more accurate assessment of prognostic factors, functional recovery, and disability. Collecting detailed information on weapon type, ballistic characteristics, and prehospital care would further strengthen the contextual interpretation of injury mechanisms and outcomes. Finally, although the models reported AUC values, Hosmer–Lemeshow statistics, and classification accuracy, internal validation measures such as bootstrap resampling or optimism‑corrected performance were not performed because these procedures are not available in standard SPSS logistic regression output. As such, the predictive performance estimates may be optimistic. Therefore, future studies should consider assessing the external validation of these estimates.

Taken together, these strengths and limitations highlight both the novelty and the challenges of conducting trauma research in conflict‑affected settings. Despite these constraints, the study provides robust multicenter evidence that fills a critical gap in the literature and establishes a foundation for future prospective registries, long‑term outcome studies, and policy interventions tailored to the realities of Palestine and comparable regions.

## Conclusion

The findings reveal a predominance of extremity injuries, yet show that thoracoabdominal and vascular trauma were associated with disproportionately severe outcomes, including higher ICU utilization, transfusion requirements, and functional dependence at discharge. Despite relatively low in‑hospital mortality, the high burden of disability underscores the need for expanded orthopedic, vascular, and critical care capacity, alongside structured rehabilitation services. By filling a critical gap in the literature, this study establishes a foundation for trauma system preparedness, evidence‑based resource allocation, and policy development in Palestine, while contributing to the global understanding of firearm injuries in conflict‑affected healthcare systems. These associations should be interpreted cautiously, particularly regarding exploratory regression findings, but they nonetheless highlight important priorities for strengthening trauma care.

## Supporting information

S1 Table Adherence to the STROBE Statement. (DOCX)

S2 Table Data collection form. (DOCX)

S3 Table A detailed breakdown of injury distribution by anatomical site. (DOCX)

S4 Table A detailed breakdown of associated injuries. (DOCX)

S5 Table A detailed breakdown of surgical interventions. (DOCX)

S6 Table Univariate analysis of factors associated with ICU admission. (DOCX)

S7 Table Univariate analysis of factors associated with mortality outcome. (DOCX)

S1 Fig Full distribution of patients across age groups. (TIF)

S1 Data Anonymized raw data. (XLSX)

## Abbreviations

ACC: American College of Cardiology
AHA: American Heart Association
CI: confidence interval
DALYs: disability-adjusted life years
EMRs: electronic medical records
GCS: Glasgow Coma Scale
ICU: intensive care unit
OR: odds ratio
Q1: first quartile
Q3: third quartile
SE: standard error
STROBE: Strengthening the Reporting of Observational Studies in Epidemiology
