# Supplementary material for: Firearm injuries in a conflict affected region: A multicenter study of epidemiological trends, injury patterns, management, and outcomes in the West Bank of Palestine
Source: PLoS One. 2026 Sep 22;21(9):e0358566. doi: 10.1371/journal.pone.0358566 (PMC13596805; doi:10.1371/journal.pone.0358566)
Supplement: Supplementary file 2 [file pone.0358566.s002.docx]

**S2 Table.** The data collection form

**Section 1: Demographics**

**Patient ID:** __________

**Gender:** ☐ Male ☐ Female

**Age (years):** __________

**Place of residence:** ☐ City ☐ Village ☐ Refugee Camp ☐ Undisclosed

**Section 2: Clinical Presentation at Emergency Room**

**Blood pressure:** ☐ Normotensive ☐ Hypotensive ☐ Hypertensive ☐ Non-recordable

**Heart rate:** ☐ Normal ☐ Bradycardia ☐ Tachycardia ☐ Non-recordable

**Glasgow Coma Scale (GCS):** ☐ >13 ☐ 9–12 ☐ 3–8 ☐ Not recorded

**Hemodynamic stability:** ☐ Stable ☐ Unstable

**Prehospital care received:** ☐ None ☐ First aid ☐ Care in another hospital/center

**Section 3: Anatomical Distribution of Injuries**

*(tick all that apply)*

**Head/Neck:** ☐ Head ☐ Face ☐ Neck

**Chest:** ☐ Chest ☐ Upper back ☐ Lower back ☐ Flank (R/L)

**Trunk:** ☐ Abdomen (RUQ, LUQ, LLQ, any) ☐ Pelvis (R/L) ☐ Groin/genital region

**Upper limb:** ☐ Right shoulder ☐ Right arm ☐ Right elbow ☐ Right forearm ☐ Right hand/wrist ☐ Left shoulder ☐ Left arm ☐ Left elbow ☐ Left forearm ☐ Left hand/wrist

**Lower limb:** ☐ Right thigh ☐ Right knee ☐ Right lower leg ☐ Right ankle/foot ☐ Left thigh ☐ Left knee ☐ Left lower leg ☐ Left ankle/foot

**Unspecified site:** ☐ Yes

**Section 4: Associated Injuries**

*(tick all that apply)*

**Fractures:** ☐ Femoral ☐ Tibial ☐ Pelvic ☐ Upper limb ☐ Foot/ankle ☐ Vertebral ☐ Thoracic girdle ☐ Skull ☐ Facial bones ☐ Hand/finger

**Vascular injury:** ☐ Yes

**Nerve injury:** ☐ Yes

**Thoracic injury:** ☐ Lung ☐ Pneumothorax/hemothorax ☐ Rib fracture ☐ Subcutaneous emphysema ☐ Mediastinal hematoma

**Cranial/intracranial injury:** ☐ Brain ☐ Intracranial hemorrhage ☐ Skull fracture

**Neck injury:** ☐ Soft tissue ☐ Laryngeal ☐ Vertebral artery ☐ Major vein ☐ Spinal cord

**Abdominal injury:** ☐ Solid organ (liver, spleen, kidney) ☐ Hollow viscus (stomach, small intestine, colon, duodenum, appendix, cecum) ☐ Major abdominal vascular

**Pelvic/retroperitoneal hematoma:** ☐ Yes

**Genitourinary injury:** ☐ Bladder ☐ Ureter ☐ Urethra ☐ Rectum ☐ Scrotum ☐ Testis ☐ Penis

**Other soft tissue/muscle/tendon injuries:** ☐ Yes

**Section 5: Surgical Interventions**

*(tick all that apply)*

**Wound management:** ☐ Dressing ☐ Debridement ☐ Hemostasis/closure ☐ Exploration

**Bullet/foreign body removal:** ☐ Yes

**Grafting:** ☐ Skin ☐ Bone

**Fracture stabilization/fixation:** ☐ External fixation ☐ ORIF ☐ Cast ☐ Splint ☐ Intramedullary nailing ☐ Skeletal traction ☐ Other

**Exploratory surgery:** ☐ Laparotomy ☐ Retroperitoneal ☐ Neck exploration

**Thoracic surgery:** ☐ Thoracotomy ☐ VATS

**Vascular surgery:** ☐ Exploration ☐ Repair ☐ Ligation ☐ Anastomosis ☐ Graft ☐ Bypass

**Endoscopic procedures:** ☐ Bronchoscopy ☐ Upper GI endoscopy

**Fasciotomy:** ☐ Yes

**Neurosurgery:** ☐ Craniotomy ☐ Hemicraniectomy ☐ Hematoma evacuation

**Tracheostomy:** ☐ Yes

**Ocular evisceration:** ☐ Yes

**Abdominal visceral surgery:** ☐ Appendectomy ☐ Nephrectomy ☐ Splenectomy ☐ Colostomy ☐ Diaphragmatic repair ☐ Bowel resection ☐ Primary bowel repair ☐ Liver ☐ Gastric

**Genitourinary/scrotal surgery:** ☐ Orchiectomy ☐ Reconstruction ☐ Orchiopexy ☐ Exploration

**Tendon/nerve surgery:** ☐ Tendon repair ☐ Nerve repair/graft

**Maxillofacial surgery:** ☐ Yes

**Other interventions:** __________

**Section 6: Clinical Management and Outcomes**

**ICU admission:** ☐ Yes ☐ No

**Blood transfusion:** ☐ Yes ☐ No

**Antibiotic administration:** ☐ Yes ☐ No

**Number of surgeries:** ☐ None ☐ 1 ☐ 2 ☐ ≥3

**Length of hospital stay (days):** __________

**Discharge status:** ☐ Home ☐ Referral ☐ Died ☐ Left against medical advice

**Functional outcome at discharge:** ☐ Independent ☐ Dependent ☐ Dead
