# Supplementary material for: Firearm injuries in a conflict affected region: A multicenter study of epidemiological trends, injury patterns, management, and outcomes in the West Bank of Palestine
Source: PLoS One. 2026 Sep 22;21(9):e0358566. doi: 10.1371/journal.pone.0358566 (PMC13596805; doi:10.1371/journal.pone.0358566)
Supplement: Supplementary file 3 [file pone.0358566.s003.docx]

**S3 Table.** A detailed breakdown of injury distribution by anatomical site

| **Anatomical site** | **n (%)** |
| --- | --- |
| **Head and neck (total)** | **19 (4.8)** |
| Head or face | 14 (3.6) |
| Neck | 5 (1.3) |
| **Chest** | **41 (10.5)** |
| **Trunk (thoracoabdominal & pelvic)** | **136 (34.7)** |
| Abdomen any location | 30 (7.7) |
| Right upper quadrant of abdomen | 4 (1.0) |
| Left upper quadrant of abdomen | 2 (0.5) |
| Left lower quadrant of abdomen | 5 (1.3) |
| Upper back | 14 (3.6) |
| Lower back | 14 (3.6) |
| Right flank | 3 (0.8) |
| Left flank | 4 (1.0) |
| Right pelvis | 6 (1.5) |
| Left pelvis | 4 (1.0) |
| Groin or genital region | 9 (2.3) |
| ***Upper limb (any side)*** | ***76 (19.4)*** |
| **Right upper limb (total)** | **34 (8.7)** |
| Right shoulder | 10 (2.6) |
| Right upper arm | 8 (2.0) |
| Right elbow | 4 (1.0) |
| Right proximal forearm | 3 (0.8) |
| Right forearm any location | 2 (0.5) |
| Right hand or wrist | 7 (1.8) |
| **Left upper limb (total)** | **42 (10.7)** |
| Left shoulder | 8 (2.0) |
| Left upper arm | 5 (1.3) |
| Left elbow | 8 (2.0) |
| Left distal forearm | 1 (0.3) |
| Left forearm any location | 11 (2.8) |
| Left hand or wrist | 9 (2.3) |
| ***Lower limb (any side)*** | ***265 (67.6)*** |
| **Right lower limb (total)** | **140 (35.7)** |
| Right hip | 1 (0.3) |
| Right proximal thigh | 4 (1.0) |
| Right distal thigh | 3 (0.8) |
| Right thigh any location | 57 (14.5) |
| Right knee | 18 (4.6) |
| Right lower leg | 49 (12.5) |
| Right ankle or foot | 8 (2.0) |
| **Left lower limb (total)** | **125 (31.9)** |
| Left hip | 4 (1.0) |
| Left proximal thigh | 13 (3.3) |
| Left distal thigh | 4 (1.0) |
| Left thigh any location | 29 (7.4) |
| Left knee | 16 (4.1) |
| Left lower leg | 48 (12.2) |
| Left ankle or foot | 11 (2.8) |
| **Unspecified** | **4 (1.0)** |
