# Supplementary material for: Firearm injuries in a conflict affected region: A multicenter study of epidemiological trends, injury patterns, management, and outcomes in the West Bank of Palestine
Source: PLoS One. 2026 Sep 22;21(9):e0358566. doi: 10.1371/journal.pone.0358566 (PMC13596805; doi:10.1371/journal.pone.0358566)
Supplement: Supplementary file 4 [file pone.0358566.s004.docx]

**S4 Table.** A detailed breakdown of associated injuries

| **Associated injury** | **n (%)** |
| --- | --- |
| **Soft tissue injury** | **58 (14.8)** |
| **Vascular injury** | **54 (13.8)** |
| **Nerve injury** | **15 (3.8)** |
| **Thoracic injury (any)** | **39 (9.9)** |
| Lung injury | 19 (4.8) |
| Pneumothorax or hemothorax | 10 (2.6) |
| Rib fracture | 5 (1.3) |
| Subcutaneous emphysema | 4 (1.0) |
| Mediastinal hematoma | 1 (0.3) |
| **Cranial and intracranial injury (any)** | **9 (2.3)** |
| Brain or intracranial injury | 4 (1.0) |
| Intracranial hemorrhage | 3 (0.8) |
| Skull fracture | 2 (0.5) |
| **Facial bone fracture (any)** | **7 (1.8)** |
| Facial bone fracture | 1 (0.3) |
| Zygomatic bone fracture | 1 (0.3) |
| Ethmoid bone fracture | 1 (0.3) |
| Sphenoid bone fracture | 1 (0.3) |
| Maxillary bone fracture | 1 (0.3) |
| Temporal bone fracture | 1 (0.3) |
| Nasal bone fracture | 1 (0.3) |
| **Ruptured globe** | **2 (0.5)** |
| **Neck injury (any)** | **6 (1.5)** |
| Neck soft tissue injury | 1 (0.3) |
| Laryngeal injury | 1 (0.3) |
| Vertebral artery injury | 1 (0.3) |
| Major neck vein injury | 2 (0.5) |
| Spinal cord injury | 1 (0.3) |
| **Vertebral column fracture (any)** | **8 (2.0)** |
| Vertebral fracture | 7 (1.8) |
| Spinous process fracture | 1 (0.3) |
| **Thoracic girdle fracture (any)** | **12 (3.1)** |
| Scapular fracture | 8 (2.0) |
| Clavicle fracture | 2 (0.5) |
| Sternum or manubrium fracture | 2 (0.5) |
| **Diaphragmatic injury** | **6 (1.5)** |
| **Abdominal solid organ injury (any)** | **25 (6.4)** |
| Liver injury | 13 (3.3) |
| Splenic injury | 5 (1.3) |
| Kidney injury | 7 (1.8) |
| **Hollow viscus gastrointestinal injury (any)** | **45 (11.5)** |
| Stomach injury | 9 (2.3) |
| Small intestine injury | 17 (4.3) |
| Colon injury | 16 (4.1) |
| Duodenal injury | 1 (0.3) |
| Appendiceal injury | 1 (0.3) |
| Cecal perforation | 1 (0.3) |
| **Major abdominal vascular injury (any)** | **3 (0.8)** |
| Mesenteric vascular injury | 2 (0.5) |
| Inferior vena cava injury | 1 (0.3) |
| **Pelvic / retroperitoneal hematoma (any)** | **8 (2.0)** |
| Pelvic hematoma | 4 (1.0) |
| Retroperitoneal hematoma | 4 (1.0) |
| **Genitourinary injury (any)** | **17 (4.3)** |
| Bladder injury | 3 (0.8) |
| Ureteral injury | 4 (1.0) |
| Urethral injury | 3 (0.8) |
| Rectal injury | 4 (1.0) |
| Perineal wound | 1 (0.3) |
| Scrotal injury | 4 (1.0) |
| Testicular rupture | 1 (0.3) |
| Penile glans injury | 2 (0.5) |
| **ALL fractures (any location)** | **152 (38.8)** |
| **Pelvic fracture (any)** | **17 (4.3)** |
| Pelvic ring fracture | 2 (0.5) |
| Iliac bone fracture | 8 (2.0) |
| Sacral fracture | 3 (0.8) |
| Coccygeal fracture | 1 (0.3) |
| Acetabular fracture | 2 (0.5) |
| Ischial fracture | 1 (0.3) |
| **Femoral fracture (any)** | **32 (8.2)** |
| Femoral shaft fracture | 26 (6.6) |
| Femoral neck fracture | 3 (0.8) |
| Subtrochanteric femur fracture | 3 (0.8) |
| **Tibial fracture (any)** | **38 (9.7)** |
| Tibial fracture | 17 (4.3) |
| Tibial shaft fracture | 3 (0.8) |
| Distal tibial pilon fracture | 4 (1.0) |
| Combined tibial and fibular fracture | 14 (3.6) |
| **Foot and ankle fracture (any)** | **11 (2.8)** |
| Ankle fracture | 2 (0.5) |
| Calcaneal fracture | 3 (0.8) |
| Talus fracture | 1 (0.3) |
| Cuboid fracture | 1 (0.3) |
| Metatarsal fracture | 1 (0.3) |
| Medial cuneiform fracture | 1 (0.3) |
| **Upper limb fracture (any)** | **21 (5.4)** |
| Olecranon fracture | 2 (0.5) |
| Humeral fracture | 8 (2.0) |
| Radial fracture | 5 (1.3) |
| Ulnar fracture | 3 (0.8) |
| Combined radial and ulnar fracture | 3 (0.8) |
| **Hand injury (any)** | **2 (0.5)** |
| Finger phalanx fracture | 1 (0.3) |
| Finger amputation | 1 (0.3) |
| **Tendon injury (any)** | **4 (1.0)** |
| Tendon rupture | 3 (0.8) |
| Achilles tendon rupture | 1 (0.3) |
| **Muscle injury** | **9 (2.3)** |
