# Supplementary material for: Firearm injuries in a conflict affected region: A multicenter study of epidemiological trends, injury patterns, management, and outcomes in the West Bank of Palestine
Source: PLoS One. 2026 Sep 22;21(9):e0358566. doi: 10.1371/journal.pone.0358566 (PMC13596805; doi:10.1371/journal.pone.0358566)
Supplement: Supplementary file 5 [file pone.0358566.s005.docx]

**S5 Table.** A detailed breakdown of surgical interventions

| **Surgical intervention** | **n (%)** |
| --- | --- |
| **Wound management (any)** | **136 (34.7)** |
| Wound dressing and care | 66 (16.8) |
| Surgical debridement | 42 (10.7) |
| Hemostasis with primary wound closure | 6 (1.5) |
| Wound exploration | 22 (5.6) |
| **Bullet or foreign body removal** | **44 (11.2)** |
| **Grafting procedures (any)** | **6 (1.5)** |
| Skin grafting | 5 (1.3) |
| Bone grafting | 1 (0.3) |
| **Fracture stabilization/fixation (any)** | **116 (29.6)** |
| External fixation | 63 (16.1) |
| Open reduction and internal fixation | 8 (2.0) |
| Cast application | 7 (1.8) |
| Skeletal traction | 2 (0.5) |
| Intramedullary nailing | 12 (3.1) |
| Backslab splint application | 17 (4.3) |
| Dynamic hip screw fixation | 1 (0.3) |
| Closed reduction and fixation | 2 (0.5) |
| Orthopedic joint fixation | 4 (1.0) |
| **Exploratory surgery (any)** | **60 (15.3)** |
| Exploratory laparotomy | 56 (14.3) |
| Retroperitoneal exploration | 1 (0.3) |
| Neck exploration and dissection | 3 (0.8) |
| **Thoracic surgery (any)** | **17 (4.3)** |
| Thoracotomy | 15 (3.8) |
| Video-assisted thoracoscopic surgery | 2 (0.5) |
| **Vascular surgery (any)** | **64 (16.3)** |
| Vascular exploration | 8 (2.0) |
| Vascular repair | 30 (7.7) |
| Thrombectomy | 1 (0.3) |
| Vessel ligation | 10 (2.6) |
| Vascular anastomosis | 5 (1.3) |
| Interposition vascular graft | 5 (1.3) |
| Vascular bypass | 3 (0.8) |
| Great saphenous vein graft | 2 (0.5) |
| **Endoscopic procedures (any)** | **4 (1.0)** |
| Bronchoscopy | 2 (0.5) |
| Upper gastrointestinal endoscopy | 2 (0.5) |
| **Fasciotomy** | **8 (2.0)** |
| **Neurosurgical intervention (any)** | **5 (1.3)** |
| Craniotomy | 1 (0.3) |
| Decompressive hemicraniectomy | 2 (0.5) |
| Intracranial hematoma evacuation | 2 (0.5) |
| **Tracheostomy** | **1 (0.3)** |
| **Ocular evisceration** | **1 (0.3)** |
| **Abdominal visceral surgery (any)** | **44 (11.2)** |
| Appendectomy | 1 (0.3) |
| Nephrectomy | 5 (1.3) |
| Splenectomy | 4 (1.0) |
| Colostomy stoma creation | 8 (2.0) |
| Diaphragmatic repair | 8 (2.0) |
| Bowel resection with anastomosis | 16 (4.1) |
| Primary bowel repair | 10 (2.6) |
| Liver surgery | 5 (1.3) |
| Gastric surgery | 6 (1.5) |
| **Genitourinary/scrotal surgery (any)** | **11 (2.8)** |
| Orchiectomy | 2 (0.5) |
| Urologic reconstruction | 6 (1.5) |
| Orchiopexy | 1 (0.3) |
| Testicular exploration | 2 (0.5) |
| **Tendon/nerve surgery (any)** | **13 (3.3)** |
| Tendon repair | 7 (1.8) |
| Nerve repair or grafting | 6 (1.5) |
| **Maxillofacial surgery** | **2 (0.5)** |
| **Other surgical intervention** | **9 (2.3)** |
