# Supplementary material for: Firearm injuries in a conflict affected region: A multicenter study of epidemiological trends, injury patterns, management, and outcomes in the West Bank of Palestine
Source: PLoS One. 2026 Sep 22;21(9):e0358566. doi: 10.1371/journal.pone.0358566 (PMC13596805; doi:10.1371/journal.pone.0358566)
Supplement: Supplementary file 6 [file pone.0358566.s006.docx]

**S6 Table.** Univariate analysis of factors associated with ICU admission

|  |  | **ICU admission** | |  |
| --- | --- | --- | --- | --- |
| **Variable** | **Subcategory** | **No** | **Yes** | **p-value** |
| Heart rate on arrival (beat/min) | median [Q1, Q3] | 87.0 [78.0, 93.0] | 100.0 [87.0, 122.0] | < 0.001 |
| Chest injury | No, n (%) | 288 (73.5) | 63 (16.1) | < 0.001 |
|  | Yes, n (%) | 23 (5.9) | 18 (4.6) |  |
| Abdomen (any) | No, n (%) | 297 (75.8) | 65 (16.6) | < 0.001 |
|  | Yes, n (%) | 14 (3.6) | 16 (4.1) |  |

ICU: intensive care unit, Q1: first quartile, Q3: third quartile
