# Supplementary material for: Firearm injuries in a conflict affected region: A multicenter study of epidemiological trends, injury patterns, management, and outcomes in the West Bank of Palestine
Source: PLoS One. 2026 Sep 22;21(9):e0358566. doi: 10.1371/journal.pone.0358566 (PMC13596805; doi:10.1371/journal.pone.0358566)
Supplement: Supplementary file 7 [file pone.0358566.s007.docx]

**S6 Table.** Univariate analysis of factors associated with mortality outcome

|  |  | **Mortality outcome** | |  |
| --- | --- | --- | --- | --- |
| **Variable** | **Subcategory** | **Discharged dead** | **Discharged alive** | **p-value** |
| Age (years) | median [Q1, Q3] | 30.5 [29.0, 35.0] | 19.0 [17.0, 26.0] | 0.004 |
| Heart rate on arrival (beat/min) | median [Q1, Q3] | 113.5 [112.0, 130.0] | 87.0 [79.0, 99.0] | 0.002 |

ICU: intensive care unit, Q1: first quartile, Q3: third quartile
