# Supplementary material for: Firearm injuries in a conflict affected region: A multicenter study of epidemiological trends, injury patterns, management, and outcomes in the West Bank of Palestine
Source: PLoS One. 2026 Sep 22;21(9):e0358566. doi: 10.1371/journal.pone.0358566 (PMC13596805; doi:10.1371/journal.pone.0358566)
Supplement: Supplementary file 8 [file pone.0358566.s011.docx]

#### MANUSCRIPT ID/NUMBER: PONE-D-26-16621

#### MANUSCRIPT TITLE: Firearm injuries in a conflict affected region: A multicenter study of epidemiological trends, injury patterns, management, and outcomes in the West Bank of Palestine

#### JOURNAL: PLOS One

#### EDITOR: Prof. Dr. Pawan Acharya, PhD, MSPH

**Dear editor,**

Many thanks for sharing your comments and comments of the reviewers on our manuscript submitted for publication in your highly esteemed journal. We are very delighted to learn that you and the reviewers have found the manuscript to be relevant, interesting, and consideration worthy. We are also thankful for all the insightful and constructive comments which we believe have improved the quality, clarity, and flow of the revised manuscript.

Please note that we have found all comments and suggestions provided by the reviewers as relevant and consideration worthy. In this revision, we have addressed all the comments and suggestions raised by the reviewers and the manuscript was amended accordingly. Please find below a point-to-point detailed account of responses to reviewers’ comments and how the manuscript was revised accordingly. For your convenience and to expedite the decision, all revisions and amendments were highlighted in red font.

**EDITORIAL COMMENTS**

1. **Comment:** The reviewers have identified several important issues that require careful attention, particularly regarding the interpretation of the regression analyses, the limited number of mortality events, and clarification of the analytical approach. I encourage the authors to address each reviewer comment comprehensively. Please address all reviewer comments on a point-by-point basis and clearly indicate the corresponding revisions made in the manuscript.

**Response:** We would like to sincerely thank you for the time and effort devoted to reading, reviewing, commenting on our manuscript, and for finding suitable reviewers to provide their valuable feedback. We are very grateful for the constructive and helpful comments, which we found highly insightful.

Please note that all comments raised by you and the reviewers have been carefully addressed in this revised version, and we believe that the changes have improved the quality, focus, and clarity of the manuscript. For ease of review, all revisions and amendments have been highlighted in red font in the revised manuscript. Please see below how all your comments and the comments of the reviewers were addressed.

2. **Comment:** More broadly, I believe the principal contribution of this manuscript lies in its descriptive epidemiology rather than in the multivariable prediction models. Strengthening the presentation and discussion of the descriptive findings, while presenting the regression analyses more cautiously and acknowledging their limitations, would improve the overall impact and credibility of the manuscript.

**Response:** Many thanks for this comment. We fully agree with your observation that the principal contribution of this manuscript lies in its descriptive epidemiology rather than in the multivariable prediction models. As you have suggested, we have improved the focus of the presentation by placing greater emphasis on the descriptive epidemiological findings. The presentation and discussion of descriptive results have been strengthened, while the regression analyses are now presented more cautiously with explicit acknowledgment of their limitations as you and the reviewers have suggested. We believe these revisions have enhanced the overall impact and credibility of the manuscript. Please see **Lines 95-106, 303-322, and 400-436.**

3. **Comment:** Clarify the multivariable model-building strategy and rationale for variable selection.

**Response:** Many thanks for this comment. As you have suggested, we clarified the multivariable model‑building strategy and provided a clear rationale for variable selection. Specifically, we explained that candidate variables were chosen based on their statistical significance in bivariate analyses and their clinical relevance as supported by prior literature. We also clarified why certain variables were included and why others were not, ensuring transparency in the modeling process. Please see **Lines 202-215**.

4. **Comment:** Given the novelty of this multicenter cohort, consider emphasizing the descriptive epidemiologic findings—which represent the principal contribution of the study—while presenting the regression analyses more cautiously.

**Response:** Many thanks for this comment. We fully agree that the novelty of this multicenter cohort lies primarily in its descriptive epidemiologic findings. As you have suggested, we have emphasized these descriptive results more prominently, as they represent the principal contribution of the study in this revision. Please see **Lines 303-322.**

At the same time, the regression analyses are now presented more cautiously, with explicit acknowledgment of their limitations. This balanced approach ensures that the descriptive epidemiology is highlighted while the regression findings are contextualized appropriately. Please see **Lines 400-436**.

5. **Comment:** Because mortality was uncommon, avoid overinterpreting classification accuracy and provide more informative measures of model performance if predictive analyses remain a major focus.

**Response:** We sincerely thank you and reviewers for this constructive observation. We fully acknowledge that mortality was uncommon in our cohort, and therefore the predictive analyses must be interpreted with caution. In the revised manuscript, we avoided overinterpreting classification accuracy and instead provided more informative measures of model performance, including AUC values, to better contextualize the regression results. Please see **Lines 303-322.**

These revisions ensure that the predictive analyses are presented more cautiously and transparently, in line with the limitations of the dataset. Please see **Lines 400-436.**

**REVIEWER 1:**

1. **Comment:** This manuscript addresses an important public health and trauma-care issue and provides valuable multicenter data from a region where firearm injury research remains limited. The multicenter design, relatively large sample size, inclusion of multiple hospitals, and availability of anonymized raw data are important strengths. The study has the potential to make a meaningful contribution to the literature after revision. Dear authors: This manuscript addresses an important public health and trauma-care issue and provides valuable multicenter data from a region where firearm injury research remains limited. The multicenter design, relatively large sample size, inclusion of multiple hospitals, and availability of anonymized raw data are important strengths. The study has the potential to make a meaningful contribution to the literature after revision.

**Response:** We would like to sincerely thank you for the time and effort devoted to reading, reviewing, and commenting on our manuscript. We are delighted to learn that you have found the manuscript important, interesting, and addressing a significant public health and trauma‑care issue by providing valuable multicenter data from a region where firearm injury research remains limited.

We are also grateful for highlighting the strengths of the work, including the multicenter design, relatively large sample size, inclusion of multiple hospitals, and availability of anonymized raw data. We greatly appreciate your encouraging words and recommendation for acceptance after addressing the comments.

All comments have been carefully considered and addressed in this revised version, which we believe has improved the quality, clarity, and focus of the manuscript. For ease of review, all changes have been highlighted in red font. **Please see below**.

**Major comments:**

2. **Comment:** There is a major inconsistency in the mortality regression model. The manuscript reports odds ratios below 1 for both age and heart rate (OR=0.93 for each), yet interprets these variables as predictors of increased mortality. Please verify outcome coding, regression coefficients, odds ratios, confidence intervals, and interpretation throughout the manuscript.

**Response:** Many thanks for this comment. To ensure accuracy, we sought professional statistical advice and carefully re‑examined the regression coding, coefficients, odds ratios, and confidence intervals. As part of this process, we conducted univariate analyses and added them as supplementary files (S6 and S7 Tables) to provide clearer insight into the associations. These analyses confirmed that patients who died were significantly older and had higher heart rates on arrival, and that patients admitted to the ICU also had higher heart rates and more frequent chest and abdominal injuries. We then re‑conducted the multivariate logistic regression analyses with corrected coding and diagnostics. The text in the Results section has been revised accordingly to ensure consistency with the statistical outputs, and the interpretation of odds ratios has been revised accordingly. Please see **Lines 303-322, S6 Table, S7 Table, and Table 5.**

3. **Comment:** Only 13 deaths occurred in the study cohort. The authors should justify the modeling strategy used for mortality prediction and discuss the potential for model instability and overfitting.

**Response:** Many thanks for this comment. As you have suggested, we have now explicitly cautioned that the mortality model should be considered exploratory due to the small number of events, and that the findings may be subject to model instability and overfitting. To further strengthen transparency, we added AUC values to provide more informative measures of model performance beyond classification accuracy. Please see **Lines 303-322**.

In addition, a dedicated limitation has been added to the Discussion section to discuss this. Please see **Lines 400-436.**

4. **Comment:** The study does not include standardized injury severity measures such as ISS, AIS, RTS, or TRISS. This limitation should be acknowledged more explicitly, and the potential impact on multivariable analyses should be discussed.

**Response:** Many thanks for this comment. As you have suggested, we have now explicitly discussed this in the limitations. In addition, we also discussed the impact of these on the results. Please see **Lines 417-422**.

5. **Comment:** Functional dependence at discharge is reported as a major outcome; however, no clear definition or measurement instrument is provided. The authors should clarify how functional status was assessed and categorized.

**Response:** Many thanks for this comment. As you have suggested, we have now explicitly added a definition of this outcome. Again, the limitation of this measure was also discussed in the limitations. Please see **Lines 171-178.**

6. **Comment:** Information regarding the intent and mechanism of firearm injury (conflict-related, assault, accidental, self-inflicted, etc.) is absent. This substantially limits interpretation of the epidemiological findings and should be discussed as an important limitation.

**Response:** We thank the reviewer for this important observation. Indeed, the study was conducted in a conflict‑affected area, and the injuries were overwhelmingly conflict‑related. However, to avoid political polarization and maintain a neutral scientific tone, we did not categorize cases by intent in the main analyses. To address this, we have added a dedicated limitation in the Discussion section acknowledging the absence of intent and mechanism data, and we recommend that future studies incorporate standardized documentation of injury intent to strengthen interpretation of epidemiological findings. Please see **Lines 422-427**.

7. **Comment:** The rationale for classifying blood pressure according to ACC/AHA hypertension thresholds in an acute trauma population should be clarified. These categories may not be clinically meaningful in the context of firearm trauma.

**Response:** Many thanks for this comment. As you have suggested, the reasons for adopting these thresholds were added to the revised methods. Please see **Lines 154-162.**

We also added to the limitations the implications of using these thresholds. Please see **Lines 403-408**.

8. **Comment:** There appears to be inconsistency between the regression results described in the text and Table 5. Lung injury is discussed in the Results section but is not included in the final regression table.

**Response:** Many thanks for this comment. As you have suggested, univariate analysis was conducted in this revision. Again, the multivariate regression analysis was also re-conducted. The Results section has been harmonized with Table 5, ensuring that only variables retained in the final multivariate model are described in the text. Please see **Lines 315-322**.

9. **Comment:** Several recommendations regarding expansion of ICU capacity, blood bank resources, and surgical infrastructure extend beyond the data directly collected in the study. These statements should be moderated or better justified.

**Response:** Many thanks for this comment. As you have suggested, we have revised these discussions and better justified these recommendations. Please see **Lines 339-344, 351-355, and 362-366**.

**Minor comments:**

10. **Comment:** Clarify the definition of prehospital care categories.

**Response:** Many thanks for this comment. As you have suggested, we have added clearer definition for prehospital care categories. Please see **Lines 149-152**.

11. **Comment:** Report the extent of missing data for key variables more explicitly.

**Response:** Many thanks for this comment. As you have suggested, we have explicitly added to the tables missing variables as not documented or not recorded, etc. Please see **Lines 189-192 and Table 1**.

12. **Comment:** Consider reporting ROC/AUC and additional measures of model performance.

**Response:** Many thanks for this comment. As you have suggested, we have explicitly reported the AUC and additional measures of model performance. Please see **Lines 212-215 and 303-322**.

13. **Comment:** Avoid speculative statements regarding firearm velocity, shooting distance, or injury circumstances unless supporting data are available.

**Response:** Many thanks for this comment. As you have suggested, we have revised the discussion to avoid these speculative statements. Please see **Lines 339-344, 351-355, 363-366**.

14. **Comment:** Revise statements claiming disproportionate burden among individuals younger than 18 years, as the largest age group in the cohort was 18–29 years.

**Response:** Many thanks for this observation. As you have suggested, we have revised the text and ensured that S1 Fig was uploaded. Please see **Lines 237-243 and S1 Fig.**

**REVIEWER 2:**

1. **Comment:** Lines 151–152: Regarding the assessment of hemodynamic instability, I think it is inappropriate to include systolic blood pressure of more than 170 mmHg and a GCS score of less than 13.

**Response:** We would like to sincerely thank you for the time and effort devoted to reading, reviewing, and commenting on our manuscript. We are delighted to learn that you have found the manuscript important, interesting, and addressing a significant public health and trauma‑care issue by providing valuable multicenter data from a region where firearm injury research remains limited.

We are also grateful for highlighting the strengths of the work, including the multicenter design, relatively large sample size, inclusion of multiple hospitals, and availability of anonymized raw data. We greatly appreciate your encouraging words and recommendation for acceptance after addressing the comments.

All comments have been carefully considered and addressed in this revised version, which we believe has improved the quality, clarity, and focus of the manuscript. For ease of review, all changes have been highlighted in red font. **Please see below**.

We thank the reviewer for this valuable observation. As you have suggested, the definition of hemodynamic instability was clarified. Please see **Lines 153-166**.

2. **Comment:** Please clearly state in the Introduction what issues currently exist regarding the triage of fire-arm related trauma in Palestine. It is not clear from the main text why this study needs to examine factors associated with ICU admission and in-hospital mortality.

**Response:** Many thanks for this comment. As you have suggested, we have clearly added the rationale for this study by adding more on the challenges regarding the triage of fire-arm related trauma in Palestine. Please see **Lines XXX**.

3. **Comment:** Lines 183–188: Regarding the multivariate analysis examining factors associated with ICU admission and in-hospital mortality, it is unclear why “age,” “pulse rate on arrival,” “chest trauma,” and “abdominal trauma” were selected as covariates. Please cite the prior literature and describe the process used to select these variables. Please also explain why other variables were not selected. Also, please explain why other variables were not selected. Furthermore, while this study classified “chest trauma” and “abdominal trauma” into the same category regardless of severity or the affected organ, I think it is inappropriate to group them together in this way.

**Response:** Many thanks for this comment. As you have suggested, we have clearly added the rationale for selecting these variables. Please see **Lines 95-106**.

4. **Comment:** Supplementary Figure 1 was not included in the main text; please add it.

**Response:** Many thanks for pointing this out. As you have pointed, we have included the S1 Fig in this revision.

**COMMENTS TO THE AUTHOR**

1. Is the manuscript technically sound, and do the data support the conclusions? The manuscript must describe a technically sound piece of scientific research with data that supports the conclusions. Experiments must have been conducted rigorously, with appropriate controls, replication, and sample sizes. The conclusions must be drawn appropriately based on the data presented.

Reviewer #1: Partly

Reviewer #2: Partly

**Response:** Many thanks for this comment. By addressing your comments, we have strengthened the methodological clarity, explicitly defined variable selection, corrected inconsistencies between text and tables, and moderated speculative statements. The analyses were reconducted where needed, predictors were justified with prior literature, and missing data were transparently reported. With these revisions, the data now fully support the conclusions, and the manuscript presents a technically sound piece of research with appropriate rigor, controls, and sample size.

2. Has the statistical analysis been performed appropriately and rigorously?

Reviewer #1: No

Reviewer #2: No

**Response:** Many thanks for this comment. By addressing your comments, we have clarified the process of variable selection, justified covariates with reference to prior trauma literature. We also reconducted univariate and multivariate analyses, reported missing data transparently, and added model performance metrics including ROC curves and AUC values. These revisions strengthen the methodological rigor and ensure that the statistical analyses are appropriate, transparent, and robust.

3. Have the authors made all data underlying the findings in their manuscript fully available? The PLOS Data policy requires authors to make all data underlying the findings described in their manuscript fully available without restriction, with rare exception (please refer to the Data Availability Statement in the manuscript PDF file). The data should be provided as part of the manuscript or its supporting information, or deposited to a public repository. For example, in addition to summary statistics, the data points behind means, medians and variance measures should be available. If there are restrictions on publicly sharing data—e.g. participant privacy or use of data from a third party—those must be specified.

Reviewer #1: Yes

Reviewer #2: Yes

**Response:** We thank both reviewers for acknowledging that all data underlying the findings have been made fully available in accordance with the PLOS Data policy. We appreciate this recognition and have ensured that the dataset, supporting tables, and supplementary materials remain accessible without restriction, safeguarding transparency and reproducibility.

4. Is the manuscript presented in an intelligible fashion and written in standard English?

PLOS ONE does not copyedit accepted manuscripts, so the language in submitted articles must be clear, correct, and unambiguous. Any typographical or grammatical errors should be corrected at revision, so please note any specific errors here.

Reviewer #1: Yes

Reviewer #2: Yes

**Response:** We thank both reviewers for confirming that the manuscript is presented in an intelligible fashion and written in standard English. We appreciate this recognition and have carefully corrected typographical and grammatical errors during revision to ensure clarity, accuracy, and readability.

Once again, we would like to thank you and the reviewers for the constructive feedback and comments, which we believe have improved the quality, clarity and flow of the revised manuscript.

Having addressed all comments and revised the manuscript accordingly, we are looking forward to hearing from you soon.

Kind regards

Ramzi Shawahna
