# Supplementary material for: Firearm injuries in a conflict affected region: A multicenter study of epidemiological trends, injury patterns, management, and outcomes in the West Bank of Palestine
Source: PLoS One. 2026 Sep 22;21(9):e0358566. doi: 10.1371/journal.pone.0358566 (PMC13596805; doi:10.1371/journal.pone.0358566)
Supplement: Supplementary file 9 [file pone.0358566.s012.docx]

#### MANUSCRIPT ID/NUMBER: PONE-D-26-16621

#### MANUSCRIPT TITLE: Firearm injuries in a conflict affected region: A multicenter study of epidemiological trends, injury patterns, management, and outcomes in the West Bank of Palestine

#### JOURNAL: PLOS One

#### EDITOR: Prof. Dr. Pawan Acharya, PhD, MSPH

**Dear editor,**

We would like to sincerely thank the you and both reviewers for the time, effort, and thoughtful feedback devoted to our manuscript. We greatly appreciate the recognition of the improvements made in organization, methodological transparency, and discussion, as well as the acknowledgment of the importance of this multicenter study in documenting firearm injuries in the West Bank.

All additional comments and final edits have been carefully addressed as suggested. Specifically, the interpretation of the mortality model has been softened throughout the manuscript to emphasize its exploratory nature given the small number of deaths, and the limitations of residual confounding have been further highlighted. The discrepancy between the Results text and Table 5 regarding chest injury has been corrected, and the rationale for variable selection in the multivariable models has been clarified. We have also acknowledged the absence of standardized trauma severity scores and the lack of internal validation measures as limitations. Finally, the manuscript has undergone a careful language edit to ensure consistency in terminology and formatting.

We are grateful for the constructive guidance provided, which has helped us refine the manuscript and ensure accuracy, clarity, and transparency. Thank you once again for recognizing the importance of our work and for supporting its improvement toward publication.

Please find below a point-to-point detailed account of responses to reviewers’ comments and how the manuscript was revised accordingly. For your convenience and to expedite the decision, all revisions and amendments were highlighted in red font.

**Reviewer #1:**

1. **Comment:** Thank you for the careful revision of the manuscript. The authors have addressed many of the concerns raised in the previous review, and the manuscript has improved substantially in terms of organization, methodological transparency, and discussion. The multicenter design and the inclusion of patients from eight hospitals provide valuable information on firearm injuries in the West Bank, and the study represents an important contribution to the literature.

**Response:** We sincerely thank the reviewer for the time and effort devoted to evaluating our revised manuscript. We are delighted that the improvements in organization, methodological transparency, and discussion were recognized, and we greatly appreciate the acknowledgment of the value of our multicenter design and inclusion of patients from eight hospitals. Your encouraging feedback reinforces the importance of this study, and we are grateful for your thoughtful review.

2. **Comment:** I believe the manuscript is close to being suitable for publication. However, a few issues should still be addressed before acceptance.

**Response:** We are very delighted to learn that you will recommend the acceptance of the manuscript after addressing the final comments and suggestions. Thank you for your thoughtful evaluation and constructive guidance, which have been invaluable in refining our work. Please see below how all comments and suggestions were addressed and how the manuscript was amended accordingly.

**Major remaining concern**

3. **Comment:** The principal remaining concern relates to the multivariable regression analyses. Although the statistical methods are now more clearly described, the mortality model is based on only 13 deaths. This very small number of events substantially limits the stability of logistic regression estimates and increases the risk of overfitting. While the authors appropriately acknowledge this limitation in the Discussion, the interpretation of the mortality model should be further softened throughout the manuscript. Statements suggesting independent prediction should be presented more cautiously, emphasizing that these findings are exploratory and require external validation.

**Response:** We thank the reviewer for this important observation. We fully acknowledge that the mortality model was based on only 13 deaths, which substantially limits the stability of logistic regression estimates and increases the risk of overfitting. As you have suggested, we have carefully revised the manuscript to soften the interpretation of these results throughout. Specifically, statements that previously suggested “independent prediction” have been replaced with phrasing that emphasizes observed associations. We now consistently describe the mortality findings as exploratory and highlight the need for external validation in larger cohorts. These changes ensure that the limitations of the mortality model are appropriately reflected in both the Results and Discussion sections. Please see **Lines 341-359, 371-395, and 441-450.**

**Additional comments**

4. **Comment:** The discrepancy between the Results text and Table 5 regarding chest injury should be corrected. The Results section reports chest injury as an independent predictor of ICU admission, whereas Table 5 reports a non-significant association (OR 0.52, p = 0.163). This inconsistency must be resolved.

**Response:** Many thanks for pointing this out. In this revision, the inconsistency was resolved. Please see **Lines 33-35 and 309-312**.

5. **Comment:** The rationale for selecting variables included in the multivariable models should be described more explicitly. Although the manuscript states that variables were chosen based on clinical relevance and univariate analyses, the exact selection strategy would benefit from clearer explanation.

**Response:** Many thanks for this comment. As you have suggested, the rationale for selecting variables included in the multivariable models was described more explicitly. Please see **Lines 202-213**.

6. **Comment:** Because standardized trauma severity scores (such as ISS, AIS, RTS, or TRISS) were unavailable, the limitations of the prediction models should be emphasized once more, since residual confounding by injury severity is likely.

**Response:** Many thanks for this comment. As you have suggested, we have expanded the limitation discussing unavailability of standardized trauma severity scores (such as ISS, AIS, RTS, or TRISS) emphasizing the limitations of the prediction models as residual confounding by injury severity is likely. Please see **Lines 425-436**.

7. **Comment:** The manuscript reports AUC values, Hosmer-Lemeshow statistics, and classification accuracy, which are helpful. If feasible, the authors may also consider reporting internal validation measures (for example, bootstrap validation or optimism-corrected performance). If this is not possible, it should be acknowledged as a limitation.

**Response:** Many thanks for this comment. As SPSS was used to analyze the data, these could not be performed. As you have suggested, these were discussed in the limitations. Please see **Lines 450-455**.

8. **Comment:** The manuscript would benefit from one final careful language edit to improve consistency in terminology and remove a few minor typographical and formatting inconsistencies.

**Response:** Many thanks for this comment. The manuscript was proofread and the language was edited. Please see **Lines 341-455**.

9. **Comment:** Overall, the manuscript has improved considerably since the previous version. After addressing the points above, I believe it will be suitable for publication.

**Response:** We sincerely thank you for your time, effort, and constructive feedback. We are very pleased to learn that the manuscript has improved considerably and is now you will recommend it for publication. All remaining points have been carefully addressed, and we are grateful for the guidance that has helped us refine the work to this stage.

**Reviewer #2:**

1. **Comment:** The author has appropriately addressed my questions, and I believe the submitted paper has been suitably revised. Good job.

**Response:** We sincerely thank the reviewer for the time and effort devoted to evaluating our revised manuscript. We are delighted that the reviewer has approved all revisions and recommended the acceptance of the revised manuscript.

Once again, we would like to thank you and the reviewers for the constructive feedback and comments, which we believe have improved the quality, clarity and flow of the revised manuscript.

Having addressed all comments and revised the manuscript accordingly, we are looking forward to hearing from you soon.

Kind regards

Ramzi Shawahna
