# Supplementary material for: Firearm injuries in a conflict affected region: A multicenter study of epidemiological trends, injury patterns, management, and outcomes in the West Bank of Palestine
Source: PLoS One. 2026 Sep 22;21(9):e0358566. doi: 10.1371/journal.pone.0358566 (PMC13596805; doi:10.1371/journal.pone.0358566)
Supplement: Peer review history

# Peer review: Decision Letter 0

**DOI:** 10.1371/journal.pone.0358566.r001

**Authors:** Pawan Acharya

1 Jul 2026

Dear Dr. shawahna,

Thank you for submitting your manuscript to PLOS ONE. After careful consideration, we feel that it has merit but does not fully meet PLOS ONE’s publication criteria as it currently stands. Therefore, we invite you to submit a revised version of the manuscript that addresses the points raised during the review process.

==============================

Editorial Comments:

The reviewers have identified several important issues that require careful attention, particularly regarding the interpretation of the regression analyses, the limited number of mortality events, and clarification of the analytical approach. I encourage the authors to address each reviewer comment comprehensively. Please address all reviewer comments on a point-by-point basis and clearly indicate the corresponding revisions made in the manuscript.

More broadly, I believe the principal contribution of this manuscript lies in its descriptive epidemiology rather than in the multivariable prediction models. Strengthening the presentation and discussion of the descriptive findings, while presenting the regression analyses more cautiously and acknowledging their limitations, would improve the overall impact and credibility of the manuscript.

1. Clarify the multivariable model-building strategy and rationale for variable selection.
2. Given the novelty of this multicenter cohort, consider emphasizing the descriptive epidemiologic findings—which represent the principal contribution of the study—while presenting the regression analyses more cautiously.
3. Because mortality was uncommon, avoid overinterpreting classification accuracy and provide more informative measures of model performance if predictive analyses remain a major focus.

……………………….

Comments from Reviewer 1:

This manuscript addresses an important public health and trauma-care issue and provides valuable multicenter data from a region where firearm injury research remains limited. The multicenter design, relatively large sample size, inclusion of multiple hospitals, and availability of anonymized raw data are important strengths. The study has the potential to make a meaningful contribution to the literature after revision.

Major comments:

1. There is a major inconsistency in the mortality regression model. The manuscript reports odds ratios below 1 for both age and heart rate (OR=0.93 for each), yet interprets these variables as predictors of increased mortality. Please verify outcome coding, regression coefficients, odds ratios, confidence intervals, and interpretation throughout the manuscript.

2. Only 13 deaths occurred in the study cohort. The authors should justify the modeling strategy used for mortality prediction and discuss the potential for model instability and overfitting.

3. The study does not include standardized injury severity measures such as ISS, AIS, RTS, or TRISS. This limitation should be acknowledged more explicitly, and the potential impact on multivariable analyses should be discussed.

4. Functional dependence at discharge is reported as a major outcome; however, no clear definition or measurement instrument is provided. The authors should clarify how functional status was assessed and categorized.

5. Information regarding the intent and mechanism of firearm injury (conflict-related, assault, accidental, self-inflicted, etc.) is absent. This substantially limits interpretation of the epidemiological findings and should be discussed as an important limitation.

6. The rationale for classifying blood pressure according to ACC/AHA hypertension thresholds in an acute trauma population should be clarified. These categories may not be clinically meaningful in the context of firearm trauma.

7. There appears to be inconsistency between the regression results described in the text and Table 5. Lung injury is discussed in the Results section but is not included in the final regression table.

8. Several recommendations regarding expansion of ICU capacity, blood bank resources, and surgical infrastructure extend beyond the data directly collected in the study. These statements should be moderated or better justified.

Minor comments:

1. Clarify the definition of prehospital care categories.

2. Report the extent of missing data for key variables more explicitly.

3. Consider reporting ROC/AUC and additional measures of model performance.

4. Avoid speculative statements regarding firearm velocity, shooting distance, or injury circumstances unless supporting data are available.

5. Revise statements claiming disproportionate burden among individuals younger than 18 years, as the largest age group in the cohort was 18–29 years.

…………………..

Reviewer 2:

1. Lines 151–152: Regarding the assessment of hemodynamic instability, I think it is inappropriate to include systolic blood pressure of more than 170 mmHg and a GCS score of less than 13.

2. Please clearly state in the Introduction what issues currently exist regarding the triage of fire-arm related trauma in Palestine. It is not clear from the main text why this study needs to examine factors associated with ICU admission and in-hospital mortality.

3. Lines 183–188: Regarding the multivariate analysis examining factors associated with ICU admission and in-hospital mortality, it is unclear why “age,” “pulse rate on arrival,” “chest trauma,” and “abdominal trauma” were selected as covariates. Please cite the prior literature and describe the process used to select these variables. Please also explain why other variables were not selected. Also, please explain why other variables were not selected. Furthermore, while this study classified “chest trauma” and “abdominal trauma” into the same category regardless of severity or the affected organ, I think it is inappropriate to group them together in this way.

4. Supplementary Figure 1 was not included in the main text; please add it.

==============================

Please submit your revised manuscript by Aug 15 2026 11:59PM. If you will need more time than this to complete your revisions, please reply to this message or contact the journal office at plosone@plos.org. When you're ready to submit your revision, log on to https://www.editorialmanager.com/pone/ and select the 'Submissions Needing Revision' folder to locate your manuscript file.

- A letter that responds to each point raised by the academic editor and reviewer(s). You should upload this letter as a separate file labeled 'Response to Reviewers'.
- A marked-up copy of your manuscript that highlights changes made to the original version. You should upload this as a separate file labeled 'Revised Manuscript with Track Changes'.
- An unmarked version of your revised paper without tracked changes. You should upload this as a separate file labeled 'Manuscript'.

If you would like to make changes to your financial disclosure, please include your updated statement in your cover letter. Guidelines for resubmitting your figure files are available below the reviewer comments at the end of this letter.

If applicable, we recommend that you deposit your laboratory protocols in protocols.io to enhance the reproducibility of your results. Protocols.io assigns your protocol its own identifier (DOI) so that it can be cited independently in the future. For instructions see: https://journals.plos.org/plosone/s/submission-guidelines#loc-laboratory-protocols. Additionally, PLOS ONE offers an option for publishing peer-reviewed Lab Protocol articles, which describe protocols hosted on protocols.io. Read more information on sharing protocols at https://plos.org/protocols?utm_medium=editorial-email&utm_source=authorletters&utm_campaign=protocols.

As the corresponding author, your ORCID iD is verified in the submission system and will appear in the published article. PLOS supports the use of ORCID, and we encourage all coauthors to register for an ORCID iD and use it as well. Please encourage your coauthors to verify their ORCID iD within the submission system before final acceptance, as unverified ORCID iDs will not appear in the published article. *Only*  the individual author can complete the verification step; PLOS staff *cannot*  verify ORCID iDs on behalf of authors.

We look forward to receiving your revised manuscript.

Kind regards,

Pawan Acharya, PhD, MSPH

Academic Editor

PLOS One

Journal Requirements:

When submitting your revision, we need you to address these additional requirements.

1. Please ensure that your manuscript meets PLOS ONE's style requirements, including those for file naming. The PLOS ONE style templates can be found at

https://journals.plos.org/plosone/s/file?id=wjVg/PLOSOne_formatting_sample_main_body.pdf and

https://journals.plos.org/plosone/s/file?id=ba62/PLOSOne_formatting_sample_title_authors_affiliations.pdf

2. We note that there is identifying data in the Supporting Information file S1_Data.xlsx. Due to the inclusion of these potentially identifying data, we have removed this file from your file inventory. Prior to sharing human research participant data, authors should consult with an ethics committee to ensure data are shared in accordance with participant consent and all applicable local laws.

Data sharing should never compromise participant privacy. It is therefore not appropriate to publicly share personally identifiable data on human research participants. The following are examples of data that should not be shared:

-Name, initials, physical address

-Ages more specific than whole numbers

-Internet protocol (IP) address

-Specific dates (birth dates, death dates, examination dates, etc.)

-Contact information such as phone number or email address

-Location data

-ID numbers that seem specific (long numbers, include initials, titled “Hospital ID”) rather than random (small numbers in numerical order)

Data that are not directly identifying may also be inappropriate to share, as in combination they can become identifying. For example, data collected from a small group of participants, vulnerable populations, or private groups should not be shared if they involve indirect identifiers (such as sex, ethnicity, location, etc.) that may risk the identification of study participants.

Additional guidance on preparing raw data for publication can be found in our Data Policy (https://journals.plos.org/plosone/s/data-availability#loc-human-research-participant-data-and-other-sensitive-data) and in the following article: http://www.bmj.com/content/340/bmj.c181.long.

Please remove or anonymize all personal information age, ensure that the data shared are in accordance with participant consent, and re-upload a fully anonymized data set. Please note that spreadsheet columns with personal information must be removed and not hidden as all hidden columns will appear in the published file.

3. If the reviewer comments include a recommendation to cite specific previously published works, please review and evaluate these publications to determine whether they are relevant and should be cited. There is no requirement to cite these works unless the editor has indicated otherwise.

[Note: HTML markup is below. Please do not edit.]

Reviewers' comments:

Reviewer's Responses to Questions

**Comments to the Author**

1. Is the manuscript technically sound, and do the data support the conclusions?

Reviewer #1: Partly

Reviewer #2: Partly

**********

2. Has the statistical analysis been performed appropriately and rigorously?

Reviewer #1: No

Reviewer #2: No

**********

3. Have the authors made all data underlying the findings in their manuscript fully available?

The PLOS Data policy

Reviewer #1: Yes

Reviewer #2: Yes

**********

4. Is the manuscript presented in an intelligible fashion and written in standard English?

Reviewer #1: Yes

Reviewer #2: Yes

**********

Reviewer #1: Dear authors

This manuscript addresses an important public health and trauma-care issue and provides valuable multicenter data from a region where firearm injury research remains limited. The multicenter design, relatively large sample size, inclusion of multiple hospitals, and availability of anonymized raw data are important strengths. The study has the potential to make a meaningful contribution to the literature after revision.

Major comments:

1. There is a major inconsistency in the mortality regression model. The manuscript reports odds ratios below 1 for both age and heart rate (OR=0.93 for each), yet interprets these variables as predictors of increased mortality. Please verify outcome coding, regression coefficients, odds ratios, confidence intervals, and interpretation throughout the manuscript.

2. Only 13 deaths occurred in the study cohort. The authors should justify the modeling strategy used for mortality prediction and discuss the potential for model instability and overfitting.

3. The study does not include standardized injury severity measures such as ISS, AIS, RTS, or TRISS. This limitation should be acknowledged more explicitly, and the potential impact on multivariable analyses should be discussed.

4. Functional dependence at discharge is reported as a major outcome; however, no clear definition or measurement instrument is provided. The authors should clarify how functional status was assessed and categorized.

5. Information regarding the intent and mechanism of firearm injury (conflict-related, assault, accidental, self-inflicted, etc.) is absent. This substantially limits interpretation of the epidemiological findings and should be discussed as an important limitation.

6. The rationale for classifying blood pressure according to ACC/AHA hypertension thresholds in an acute trauma population should be clarified. These categories may not be clinically meaningful in the context of firearm trauma.

7. There appears to be inconsistency between the regression results described in the text and Table 5. Lung injury is discussed in the Results section but is not included in the final regression table.

8. Several recommendations regarding expansion of ICU capacity, blood bank resources, and surgical infrastructure extend beyond the data directly collected in the study. These statements should be moderated or better justified.

Minor comments:

1. Clarify the definition of prehospital care categories.

2. Report the extent of missing data for key variables more explicitly.

3. Consider reporting ROC/AUC and additional measures of model performance.

4. Avoid speculative statements regarding firearm velocity, shooting distance, or injury circumstances unless supporting data are available.

5. Revise statements claiming disproportionate burden among individuals younger than 18 years, as the largest age group in the cohort was 18–29 years.

Overall recommendation: Major Revision.

Reviewer #2: 1. Lines 151–152: Regarding the assessment of hemodynamic instability, I think it is inappropriate to include systolic blood pressure of more than 170 mmHg and a GCS score of less than 13.

2. Please clearly state in the Introduction what issues currently exist regarding the triage of fire-arm related trauma in Palestine. It is not clear from the main text why this study needs to examine factors associated with ICU admission and in-hospital mortality.

3. Lines 183–188: Regarding the multivariate analysis examining factors associated with ICU admission and in-hospital mortality, it is unclear why “age,” “pulse rate on arrival,” “chest trauma,” and “abdominal trauma” were selected as covariates. Please cite the prior literature and describe the process used to select these variables. Please also explain why other variables were not selected. Also, please explain why other variables were not selected. Furthermore, while this study classified “chest trauma” and “abdominal trauma” into the same category regardless of severity or the affected organ, I think it is inappropriate to group them together in this way.

4. Supplementary Figure 1 was not included in the main text; please add it.

**********

what does this mean?). If published, this will include your full peer review and any attached files.

If you choose “no”, your identity will remain anonymous but your review may still be made public.

**Do you want your identity to be public for this peer review?**  For information about this choice, including consent withdrawal, please see our Privacy Policy

Reviewer #1: No

Reviewer #2: No

**********

[NOTE: If reviewer comments were submitted as an attachment file, they will be attached to this email and accessible via the submission site. Please log into your account, locate the manuscript record, and check for the action link "View Attachments". If this link does not appear, there are no attachment files.]

To ensure your figures meet our technical requirements, please review our figure guidelines: https://journals.plos.org/plosone/s/figures

You may also use PLOS’s free figure tool, NAAS, to help you prepare publication quality figures: https://journals.plos.org/plosone/s/figures#loc-tools-for-figure-preparation.

NAAS will assess whether your figures meet our technical requirements by comparing each figure against our figure specifications.

# Author comment: Author response to Decision Letter 1

**DOI:** 10.1371/journal.pone.0358566.r002

20 Jul 2026

MANUSCRIPT ID/NUMBER: PONE-D-26-16621

MANUSCRIPT TITLE: Firearm injuries in a conflict affected region: A multicenter study of epidemiological trends, injury patterns, management, and outcomes in the West Bank of Palestine

JOURNAL: PLOS One

EDITOR: Prof. Dr. Pawan Acharya, PhD, MSPH

Dear editor,

Many thanks for sharing your comments and comments of the reviewers on our manuscript submitted for publication in your highly esteemed journal. We are very delighted to learn that you and the reviewers have found the manuscript to be relevant, interesting, and consideration worthy. We are also thankful for all the insightful and constructive comments which we believe have improved the quality, clarity, and flow of the revised manuscript.

Please note that we have found all comments and suggestions provided by the reviewers as relevant and consideration worthy. In this revision, we have addressed all the comments and suggestions raised by the reviewers and the manuscript was amended accordingly. Please find below a point-to-point detailed account of responses to reviewers’ comments and how the manuscript was revised accordingly. For your convenience and to expedite the decision, all revisions and amendments were highlighted in red font.

EDITORIAL COMMENTS

1. Comment: The reviewers have identified several important issues that require careful attention, particularly regarding the interpretation of the regression analyses, the limited number of mortality events, and clarification of the analytical approach. I encourage the authors to address each reviewer comment comprehensively. Please address all reviewer comments on a point-by-point basis and clearly indicate the corresponding revisions made in the manuscript.

Response: We would like to sincerely thank you for the time and effort devoted to reading, reviewing, commenting on our manuscript, and for finding suitable reviewers to provide their valuable feedback. We are very grateful for the constructive and helpful comments, which we found highly insightful.

Please note that all comments raised by you and the reviewers have been carefully addressed in this revised version, and we believe that the changes have improved the quality, focus, and clarity of the manuscript. For ease of review, all revisions and amendments have been highlighted in red font in the revised manuscript. Please see below how all your comments and the comments of the reviewers were addressed.

2. Comment: More broadly, I believe the principal contribution of this manuscript lies in its descriptive epidemiology rather than in the multivariable prediction models. Strengthening the presentation and discussion of the descriptive findings, while presenting the regression analyses more cautiously and acknowledging their limitations, would improve the overall impact and credibility of the manuscript.

Response: Many thanks for this comment. We fully agree with your observation that the principal contribution of this manuscript lies in its descriptive epidemiology rather than in the multivariable prediction models. As you have suggested, we have improved the focus of the presentation by placing greater emphasis on the descriptive epidemiological findings. The presentation and discussion of descriptive results have been strengthened, while the regression analyses are now presented more cautiously with explicit acknowledgment of their limitations as you and the reviewers have suggested. We believe these revisions have enhanced the overall impact and credibility of the manuscript. Please see Lines 95-106, 303-322, and 400-436.

3. Comment: Clarify the multivariable model-building strategy and rationale for variable selection.

Response: Many thanks for this comment. As you have suggested, we clarified the multivariable model‑building strategy and provided a clear rationale for variable selection. Specifically, we explained that candidate variables were chosen based on their statistical significance in bivariate analyses and their clinical relevance as supported by prior literature. We also clarified why certain variables were included and why others were not, ensuring transparency in the modeling process. Please see Lines 202-215.

4. Comment: Given the novelty of this multicenter cohort, consider emphasizing the descriptive epidemiologic findings—which represent the principal contribution of the study—while presenting the regression analyses more cautiously.

Response: Many thanks for this comment. We fully agree that the novelty of this multicenter cohort lies primarily in its descriptive epidemiologic findings. As you have suggested, we have emphasized these descriptive results more prominently, as they represent the principal contribution of the study in this revision. Please see Lines 303-322.

At the same time, the regression analyses are now presented more cautiously, with explicit acknowledgment of their limitations. This balanced approach ensures that the descriptive epidemiology is highlighted while the regression findings are contextualized appropriately. Please see Lines 400-436.

5. Comment: Because mortality was uncommon, avoid overinterpreting classification accuracy and provide more informative measures of model performance if predictive analyses remain a major focus.

Response: We sincerely thank you and reviewers for this constructive observation. We fully acknowledge that mortality was uncommon in our cohort, and therefore the predictive analyses must be interpreted with caution. In the revised manuscript, we avoided overinterpreting classification accuracy and instead provided more informative measures of model performance, including AUC values, to better contextualize the regression results. Please see Lines 303-322.

These revisions ensure that the predictive analyses are presented more cautiously and transparently, in line with the limitations of the dataset. Please see Lines 400-436.

REVIEWER 1:

1. Comment: This manuscript addresses an important public health and trauma-care issue and provides valuable multicenter data from a region where firearm injury research remains limited. The multicenter design, relatively large sample size, inclusion of multiple hospitals, and availability of anonymized raw data are important strengths. The study has the potential to make a meaningful contribution to the literature after revision. Dear authors: This manuscript addresses an important public health and trauma-care issue and provides valuable multicenter data from a region where firearm injury research remains limited. The multicenter design, relatively large sample size, inclusion of multiple hospitals, and availability of anonymized raw data are important strengths. The study has the potential to make a meaningful contribution to the literature after revision.

Response: We would like to sincerely thank you for the time and effort devoted to reading, reviewing, and commenting on our manuscript. We are delighted to learn that you have found the manuscript important, interesting, and addressing a significant public health and trauma‑care issue by providing valuable multicenter data from a region where firearm injury research remains limited.

We are also grateful for highlighting the strengths of the work, including the multicenter design, relatively large sample size, inclusion of multiple hospitals, and availability of anonymized raw data. We greatly appreciate your encouraging words and recommendation for acceptance after addressing the comments.

All comments have been carefully considered and addressed in this revised version, which we believe has improved the quality, clarity, and focus of the manuscript. For ease of review, all changes have been highlighted in red font. Please see below.

Major comments:

2. Comment: There is a major inconsistency in the mortality regression model. The manuscript reports odds ratios below 1 for both age and heart rate (OR=0.93 for each), yet interprets these variables as predictors of increased mortality. Please verify outcome coding, regression coefficients, odds ratios, confidence intervals, and interpretation throughout the manuscript.

Response: Many thanks for this comment. To ensure accuracy, we sought professional statistical advice and carefully re‑examined the regression coding, coefficients, odds ratios, and confidence intervals. As part of this process, we conducted univariate analyses and added them as supplementary files (S6 and S7 Tables) to provide clearer insight into the associations. These analyses confirmed that patients who died were significantly older and had higher heart rates on arrival, and that patients admitted to the ICU also had higher heart rates and more frequent chest and abdominal injuries. We then re‑conducted the multivariate logistic regression analyses with corrected coding and diagnostics. The text in the Results section has been revised accordingly to ensure consistency with the statistical outputs, and the interpretation of odds ratios has been revised accordingly. Please see Lines 303-322, S6 Table, S7 Table, and Table 5.

3. Comment: Only 13 deaths occurred in the study cohort. The authors should justify the modeling strategy used for mortality prediction and discuss the potential for model instability and overfitting.

Response: Many thanks for this comment. As you have suggested, we have now explicitly cautioned that the mortality model should be considered exploratory due to the small number of events, and that the findings may be subject to model instability and overfitting. To further strengthen transparency, we added AUC values to provide more informative measures of model performance beyond classification accuracy. Please see Lines 303-322.

In addition, a dedicated limitation has been added to the Discussion section to discuss this. Please see Lines 400-436.

4. Comment: The study does not include standardized injury severity measures such as ISS, AIS, RTS, or TRISS. This limitation should be acknowledged more explicitly, and the potential impact on multivariable analyses should be discussed.

Response: Many thanks for this comment. As you have suggested, we have now explicitly discussed this in the limitations. In addition, we also discussed the impact of these on the results. Please see Lines 417-422.

5. Comment: Functional dependence at discharge is reported as a major outcome; however, no clear definition or measurement instrument is provided. The authors should clarify how functional status was assessed and categorized.

Response: Many thanks for this comment. As you have suggested, we have now explicitly added a definition of this outcome. Again, the limitation of this measure was also discussed in the limitations. Please see Lines 171-178.

6. Comment: Information regarding the intent and mechanism of firearm injury (conflict-related, assault, accidental, self-inflicted, etc.) is absent. This substantially limits interpretation of the epidemiological findings and should be discussed as an important limitation.

Response: We thank the reviewer for this important observation. Indeed, the study was conducted in a conflict‑affected area, and the injuries were overwhelmingly conflict‑related. However, to avoid political polarization and maintain a neutral scientific tone, we did not categorize cases by intent in the main analyses. To address this, we have added a dedicated limitation in the Discussion section acknowledging the absence of intent and mechanism data, and we recommend that future studies incorporate standardized documentation of injury intent to strengthen interpretation of epidemiological findings. Please see Lines 422-427.

7. Comment: The rationale for classifying blood pressure according to ACC/AHA hypertension thresholds in an acute trauma population should be clarified. These categories may not be clinically meaningful in the context of firearm trauma.

Response: Many thanks for this comment. As you have suggested, the reasons for adopting these thresholds were added to the revised methods. Please see Lines 154-162.

We also added to the limitations the implications of using these thresholds. Please see Lines 403-408.

8. Comment: There appears to be inconsistency between the regression results described in the text and Table 5. Lung injury is discussed in the Results section but is not included in the final regression table.

Response: Many thanks for this comment. As you have suggested, univariate analysis was conducted in this revision. Again, the multivariate regression analysis was also re-conducted. The Results section has been harmonized with Table 5, ensuring that only variables retained in the final multivariate model are described in the text. Please see Lines 315-322.

9. Comment: Several recommendations regarding expansion of ICU capacity, blood bank resources, and surgical infrastructure extend beyond the data directly collected in the study. These statements should be moderated or better justified.

Response: Many thanks for this comment. As you have suggested, we have revised these discussions and better justified these recommendations. Please see Lines 339-344, 351-355, and 362-366.

Minor comments:

10. Comment: Clarify the definition of prehospital care categories.

Response: Many thanks for this comment. As you have suggested, we have added clearer definition for prehospital care categories. Please see Lines 149-152.

11. Comment: Report the extent of missing data for key variables more explicitly.

Response: Many thanks for this comment. As you have suggested, we have explicitly added to the tables missing variables as not documented or not recorded, etc. Please see Lines 189-192 and Table 1.

12. Comment: Consider reporting ROC/AUC and additional measures of model performance.

Response: Many thanks for this comment. As you have suggested, we have explicitly reported the AUC and additional measures of model performance. Please see Lines 212-215 and 303-322.

13. Comment: Avoid speculative statements regarding firearm velocity, shooting distance, or injury circumstances unless supporting data are available.

Response: Many thanks for this comment. As you have suggested, we have revised the discussion to avoid these speculative statements. Please see Lines 339-344, 351-355, 363-366.

14. Comment: Revise statements claiming disproportionate burden among individuals younger than 18 years, as the largest age group in the cohort was 18–29 years.

Response: Many thanks for this observation. As you have suggested, we have revised the text and ensured that S1 Fig was uploaded. Please see Lines 237-243 and S1 Fig.

REVIEWER 2:

1. Comment: Lines 151–152: Regarding the assessment of hemodynamic instability, I think it is inappropriate to include systolic blood pressure of more than 170 mmHg and a GCS score of less than 13.

Response: We would like to sincerely thank you for the time and effort devoted to reading, reviewing, and commenting on our manuscript. We are delighted to learn that you have found the manuscript important, interesting, and addressing a significant public health and trauma‑care issue by providing valuable multicenter data from a region where firearm injury research remains limited.

We are also grateful for highlighting the strengths of the work, including the multicenter design, relatively large sample size, inclusion of multiple hospitals, and availability of anonymized raw data. We greatly appreciate your encouraging words and recommendation for acceptance after addressing the comments.

All comments have been carefully considered and addressed in this revised version, which we believe has improved the quality, clarity, and focus of the manuscript. For ease of review, all changes have been highlighted in red font. Please see below.

We thank the reviewer for this valuable observation. As you have suggested, the definition of hemodynamic instability was clarified. Please see Lines 153-166.

2. Comment: Please clearly state in the Introduction what issues currently exist regarding the triage of fire-arm related trauma in Palestine. It is not clear from the main text why this study needs to examine factors associated with ICU admission and in-hospital mortality.

Response: Many thanks for this comment. As you have suggested, we have clearly added the rationale for this study by adding more on the challenges regarding the triage of fire-arm related trauma in Palestine. Please see Lines XXX.

3. Comment: Lines 183–188: Regarding the multivariate analysis examining factors associated with ICU admission and in-hospital mortality, it is unclear why “age,” “pulse rate on arrival,” “chest trauma,” and “abdominal trauma” were selected as covariates. Please cite the prior literature and describe the process used to select these variables. Please also explain why other variables were not selected. Also, please explain why other variables were not selected. Furthermore, while this study classified “chest trauma” and “abdominal trauma” into the same category regardless of severity or the affected organ, I think it is inappropriate to group them together in this way.

Response: Many thanks for this comment. As you have suggested, we have clearly added the rationale for selecting these variables. Please see Lines 95-106.

4. Comment: Supplementary Figure 1 was not included in the main text; please add it.

Response: Many thanks for pointing this out. As you have pointed, we have included the S1 Fig in this revision.

COMMENTS TO THE AUTHOR

1. Is the manuscript technically sound, and do the data support the conclusions? The manuscript must describe a technically sound piece of scientific research with data that supports the conclusions. Experiments must have been conducted rigorously, with appropriate controls, replication, and sample sizes. The conclusions must be drawn appropriately based on the data presented.

Reviewer #1: Partly

Reviewer #2: Partly

Response: Many thanks for this comment. By addressing your comments, we have strengthened the methodological clarity, explicitly defined variable selection, corrected inconsistencies between text and tables, and moderated speculative statements. The analyses were reconducted where needed, predictors were justified with prior literature, and missing data were transparently reported. With these revisions, the data now fully support the conclusions, and the manuscript presents a technically sound piece of research with appropriate rigor, controls, and sample size.

2. Has the statistical analysis been performed appropriately and rigorously?

Reviewer #1: No

Reviewer #2: No

Response: Many thanks for this comment. By addressing your comments, we have clarified the process of variable selection, justified covariates with reference to prior trauma literature. We also reconducted univariate and multivariate analyses, reported missing data transparently, and added model performance metrics including ROC curves and AUC values. These revisions strengthen the methodological rigor and ensure that the statistical analyses are appropriate, transparent, and robust.

3. Have the authors made all data underlying the findings in their manuscript fully available? The PLOS Data policy requires authors to make all data underlying the findings described in their manuscript fully available without restriction, with rare exception (please refer to the Data Availability Statement in the manuscript PDF file). The data should be provided as part of the manuscript or its supporting information, or deposited to a public repository. For example, in addition to summary statistics, the data points behind means, medians and variance measures should be available. If there are restrictions on publicly sharing data—e.g. participant privacy or use of data from a third party—those must be specified.

Reviewer #1: Yes

Reviewer #2: Yes

Response: We thank both reviewers for acknowledging that all data underlying the findings have been made fully available in accordance with the PLOS Data policy. We appreciate this recognition and have ensured that the dataset, supporting tables, and supplementary materials remain accessible without restriction, safeguarding transparency and reproducibility.

4. Is the manuscript presented in an intelligible fashion and written in standard English?

PLOS ONE does not copyedit accepted manuscripts, so the language in submitted articles must be clear, correct, and unambiguous. Any typographical or grammatical errors should be corrected at revision, so please note any specific errors here.

Reviewer #1: Yes

Reviewer #2: Yes

Response: We thank both reviewers for confirming that the manuscript is presented in an intelligible fashion and written in standard English. We appreciate this recognition and have carefully corrected typographical and grammatical errors during revision to ensure clarity, accuracy, and readability.

Once again, we would like to thank you and the reviewers for the constructive feedback and comments, which we believe have improved the quality, clarity and flow of the revised manuscript.

Having addressed all comments and revised the manuscript accordingly, we are looking forward to hearing from you soon.

Kind regards

Ramzi Shawahna

Attachment Submitted filename: 02. Response to Reviewers.docx

# Peer review: Decision Letter 1

**DOI:** 10.1371/journal.pone.0358566.r003

**Authors:** Pawan Acharya

12 Aug 2026

Dear Dr. shawahna,

Thank you for submitting your manuscript to PLOS ONE. After careful consideration, we feel that it has merit but does not fully meet PLOS ONE’s publication criteria as it currently stands. Therefore, we invite you to submit a revised version of the manuscript that addresses the points raised during the review process.

**Comments from the reviewer:**

I believe the manuscript is close to being suitable for publication. However, a few issues should still be addressed before acceptance.

Major remaining concern

The principal remaining concern relates to the multivariable regression analyses. Although the statistical methods are now more clearly described, the mortality model is based on only 13 deaths. This very small number of events substantially limits the stability of logistic regression estimates and increases the risk of overfitting. While the authors appropriately acknowledge this limitation in the Discussion, the interpretation of the mortality model should be further softened throughout the manuscript. Statements suggesting independent prediction should be presented more cautiously, emphasizing that these findings are exploratory and require external validation.

Additional comments

The discrepancy between the Results text and Table 5 regarding chest injury should be corrected. The Results section reports chest injury as an independent predictor of ICU admission, whereas Table 5 reports a non-significant association (OR 0.52, p = 0.163). This inconsistency must be resolved.

The rationale for selecting variables included in the multivariable models should be described more explicitly. Although the manuscript states that variables were chosen based on clinical relevance and univariate analyses, the exact selection strategy would benefit from clearer explanation.

Because standardized trauma severity scores (such as ISS, AIS, RTS, or TRISS) were unavailable, the limitations of the prediction models should be emphasized once more, since residual confounding by injury severity is likely.

The manuscript reports AUC values, Hosmer-Lemeshow statistics, and classification accuracy, which are helpful. If feasible, the authors may also consider reporting internal validation measures (for example, bootstrap validation or optimism-corrected performance). If this is not possible, it should be acknowledged as a limitation.

The manuscript would benefit from one final careful language edit to improve consistency in terminology and remove a few minor typographical and formatting inconsistencies.

Please submit your revised manuscript by Oct 11 2026 11:59PM. If you will need more time than this to complete your revisions, please reply to this message or contact the journal office at plosone@plos.org. When you're ready to submit your revision, log on to https://www.editorialmanager.com/pone/ and select the 'Submissions Needing Revision' folder to locate your manuscript file.

- A letter that responds to each point raised by the academic editor and reviewer(s). You should upload this letter as a separate file labeled 'Response to Reviewers'.
- A marked-up copy of your manuscript that highlights changes made to the original version. You should upload this as a separate file labeled 'Revised Manuscript with Track Changes'.
- An unmarked version of your revised paper without tracked changes. You should upload this as a separate file labeled 'Manuscript'.

If you would like to make changes to your financial disclosure, please include your updated statement in your cover letter. Guidelines for resubmitting your figure files are available below the reviewer comments at the end of this letter.

If applicable, we recommend that you deposit your laboratory protocols in protocols.io to enhance the reproducibility of your results. Protocols.io assigns your protocol its own identifier (DOI) so that it can be cited independently in the future. For instructions see: https://journals.plos.org/plosone/s/submission-guidelines#loc-laboratory-protocols. Additionally, PLOS ONE offers an option for publishing peer-reviewed Lab Protocol articles, which describe protocols hosted on protocols.io. Read more information on sharing protocols at https://plos.org/protocols?utm_medium=editorial-email&utm_source=authorletters&utm_campaign=protocols.

As the corresponding author, your ORCID iD is verified in the submission system and will appear in the published article. PLOS supports the use of ORCID, and we encourage all coauthors to register for an ORCID iD and use it as well. Please encourage your coauthors to verify their ORCID iD within the submission system before final acceptance, as unverified ORCID iDs will not appear in the published article. *Only*  the individual author can complete the verification step; PLOS staff *cannot*  verify ORCID iDs on behalf of authors.

We look forward to receiving your revised manuscript.

Kind regards,

Pawan Acharya, PhD, MSPH

Academic Editor

PLOS One

Journal Requirements:

If the reviewer comments include a recommendation to cite specific previously published works, please review and evaluate these publications to determine whether they are relevant and should be cited. There is no requirement to cite these works unless the editor has indicated otherwise.

Please review your reference list to ensure that it is complete and correct. If you have cited papers that have been retracted, please include the rationale for doing so in the manuscript text, or remove these references and replace them with relevant current references. Any changes to the reference list should be mentioned in the rebuttal letter that accompanies your revised manuscript. If you need to cite a retracted article, indicate the article’s retracted status in the References list and also include a citation and full reference for the retraction notice.

Additional Editor Comments:

Please address all comments from the reviewer on point-to-point basis.

[Note: HTML markup is below. Please do not edit.]

Reviewers' comments:

Reviewer's Responses to Questions

**Comments to the Author**

Reviewer #1: (No Response)

Reviewer #2: All comments have been addressed

**********

2. Is the manuscript technically sound, and do the data support the conclusions?

Reviewer #1: Partly

Reviewer #2: Yes

**********

3. Has the statistical analysis been performed appropriately and rigorously?

Reviewer #1: No

Reviewer #2: Yes

**********

4. Have the authors made all data underlying the findings in their manuscript fully available?

The PLOS Data policy

Reviewer #1: (No Response)

Reviewer #2: Yes

**********

5. Is the manuscript presented in an intelligible fashion and written in standard English?

Reviewer #1: Yes

Reviewer #2: Yes

**********

Reviewer #1: Thank you for the careful revision of the manuscript. The authors have addressed many of the concerns raised in the previous review, and the manuscript has improved substantially in terms of organization, methodological transparency, and discussion. The multicenter design and the inclusion of patients from eight hospitals provide valuable information on firearm injuries in the West Bank, and the study represents an important contribution to the literature.

I believe the manuscript is close to being suitable for publication. However, a few issues should still be addressed before acceptance.

Major remaining concern

The principal remaining concern relates to the multivariable regression analyses. Although the statistical methods are now more clearly described, the mortality model is based on only 13 deaths. This very small number of events substantially limits the stability of logistic regression estimates and increases the risk of overfitting. While the authors appropriately acknowledge this limitation in the Discussion, the interpretation of the mortality model should be further softened throughout the manuscript. Statements suggesting independent prediction should be presented more cautiously, emphasizing that these findings are exploratory and require external validation.

Additional comments

The discrepancy between the Results text and Table 5 regarding chest injury should be corrected. The Results section reports chest injury as an independent predictor of ICU admission, whereas Table 5 reports a non-significant association (OR 0.52, p = 0.163). This inconsistency must be resolved.

The rationale for selecting variables included in the multivariable models should be described more explicitly. Although the manuscript states that variables were chosen based on clinical relevance and univariate analyses, the exact selection strategy would benefit from clearer explanation.

Because standardized trauma severity scores (such as ISS, AIS, RTS, or TRISS) were unavailable, the limitations of the prediction models should be emphasized once more, since residual confounding by injury severity is likely.

The manuscript reports AUC values, Hosmer-Lemeshow statistics, and classification accuracy, which are helpful. If feasible, the authors may also consider reporting internal validation measures (for example, bootstrap validation or optimism-corrected performance). If this is not possible, it should be acknowledged as a limitation.

The manuscript would benefit from one final careful language edit to improve consistency in terminology and remove a few minor typographical and formatting inconsistencies.

Overall, the manuscript has improved considerably since the previous version. After addressing the points above, I believe it will be suitable for publication.

Reviewer #2: The author has appropriately addressed my questions, and I believe the submitted paper has been suitably revised. Good job.

**********

what does this mean?). If published, this will include your full peer review and any attached files.

If you choose “no”, your identity will remain anonymous but your review may still be made public.

**Do you want your identity to be public for this peer review?**  For information about this choice, including consent withdrawal, please see our Privacy Policy

Reviewer #1: No

Reviewer #2: No

**********

[NOTE: If reviewer comments were submitted as an attachment file, they will be attached to this email and accessible via the submission site. Please log into your account, locate the manuscript record, and check for the action link "View Attachments". If this link does not appear, there are no attachment files.]

To ensure your figures meet our technical requirements, please review our figure guidelines: https://journals.plos.org/plosone/s/figures

You may also use PLOS’s free figure tool, NAAS, to help you prepare publication quality figures: https://journals.plos.org/plosone/s/figures#loc-tools-for-figure-preparation.

NAAS will assess whether your figures meet our technical requirements by comparing each figure against our figure specifications.

# Author comment: Author response to Decision Letter 2

**DOI:** 10.1371/journal.pone.0358566.r004

20 Aug 2026

MANUSCRIPT ID/NUMBER: PONE-D-26-16621

MANUSCRIPT TITLE: Firearm injuries in a conflict affected region: A multicenter study of epidemiological trends, injury patterns, management, and outcomes in the West Bank of Palestine

JOURNAL: PLOS One

EDITOR: Prof. Dr. Pawan Acharya, PhD, MSPH

Dear editor,

We would like to sincerely thank the you and both reviewers for the time, effort, and thoughtful feedback devoted to our manuscript. We greatly appreciate the recognition of the improvements made in organization, methodological transparency, and discussion, as well as the acknowledgment of the importance of this multicenter study in documenting firearm injuries in the West Bank.

All additional comments and final edits have been carefully addressed as suggested. Specifically, the interpretation of the mortality model has been softened throughout the manuscript to emphasize its exploratory nature given the small number of deaths, and the limitations of residual confounding have been further highlighted. The discrepancy between the Results text and Table 5 regarding chest injury has been corrected, and the rationale for variable selection in the multivariable models has been clarified. We have also acknowledged the absence of standardized trauma severity scores and the lack of internal validation measures as limitations. Finally, the manuscript has undergone a careful language edit to ensure consistency in terminology and formatting.

We are grateful for the constructive guidance provided, which has helped us refine the manuscript and ensure accuracy, clarity, and transparency. Thank you once again for recognizing the importance of our work and for supporting its improvement toward publication.

Please find below a point-to-point detailed account of responses to reviewers’ comments and how the manuscript was revised accordingly. For your convenience and to expedite the decision, all revisions and amendments were highlighted in red font.

Reviewer #1:

1. Comment: Thank you for the careful revision of the manuscript. The authors have addressed many of the concerns raised in the previous review, and the manuscript has improved substantially in terms of organization, methodological transparency, and discussion. The multicenter design and the inclusion of patients from eight hospitals provide valuable information on firearm injuries in the West Bank, and the study represents an important contribution to the literature.

Response: We sincerely thank the reviewer for the time and effort devoted to evaluating our revised manuscript. We are delighted that the improvements in organization, methodological transparency, and discussion were recognized, and we greatly appreciate the acknowledgment of the value of our multicenter design and inclusion of patients from eight hospitals. Your encouraging feedback reinforces the importance of this study, and we are grateful for your thoughtful review.

2. Comment: I believe the manuscript is close to being suitable for publication. However, a few issues should still be addressed before acceptance.

Response: We are very delighted to learn that you will recommend the acceptance of the manuscript after addressing the final comments and suggestions. Thank you for your thoughtful evaluation and constructive guidance, which have been invaluable in refining our work. Please see below how all comments and suggestions were addressed and how the manuscript was amended accordingly.

Major remaining concern

3. Comment: The principal remaining concern relates to the multivariable regression analyses. Although the statistical methods are now more clearly described, the mortality model is based on only 13 deaths. This very small number of events substantially limits the stability of logistic regression estimates and increases the risk of overfitting. While the authors appropriately acknowledge this limitation in the Discussion, the interpretation of the mortality model should be further softened throughout the manuscript. Statements suggesting independent prediction should be presented more cautiously, emphasizing that these findings are exploratory and require external validation.

Response: We thank the reviewer for this important observation. We fully acknowledge that the mortality model was based on only 13 deaths, which substantially limits the stability of logistic regression estimates and increases the risk of overfitting. As you have suggested, we have carefully revised the manuscript to soften the interpretation of these results throughout. Specifically, statements that previously suggested “independent prediction” have been replaced with phrasing that emphasizes observed associations. We now consistently describe the mortality findings as exploratory and highlight the need for external validation in larger cohorts. These changes ensure that the limitations of the mortality model are appropriately reflected in both the Results and Discussion sections. Please see Lines 341-359, 371-395, and 441-450.

Additional comments

4. Comment: The discrepancy between the Results text and Table 5 regarding chest injury should be corrected. The Results section reports chest injury as an independent predictor of ICU admission, whereas Table 5 reports a non-significant association (OR 0.52, p = 0.163). This inconsistency must be resolved.

Response: Many thanks for pointing this out. In this revision, the inconsistency was resolved. Please see Lines 33-35 and 309-312.

5. Comment: The rationale for selecting variables included in the multivariable models should be described more explicitly. Although the manuscript states that variables were chosen based on clinical relevance and univariate analyses, the exact selection strategy would benefit from clearer explanation.

Response: Many thanks for this comment. As you have suggested, the rationale for selecting variables included in the multivariable models was described more explicitly. Please see Lines 202-213.

6. Comment: Because standardized trauma severity scores (such as ISS, AIS, RTS, or TRISS) were unavailable, the limitations of the prediction models should be emphasized once more, since residual confounding by injury severity is likely.

Response: Many thanks for this comment. As you have suggested, we have expanded the limitation discussing unavailability of standardized trauma severity scores (such as ISS, AIS, RTS, or TRISS) emphasizing the limitations of the prediction models as residual confounding by injury severity is likely. Please see Lines 425-436.

7. Comment: The manuscript reports AUC values, Hosmer-Lemeshow statistics, and classification accuracy, which are helpful. If feasible, the authors may also consider reporting internal validation measures (for example, bootstrap validation or optimism-corrected performance). If this is not possible, it should be acknowledged as a limitation.

Response: Many thanks for this comment. As SPSS was used to analyze the data, these could not be performed. As you have suggested, these were discussed in the limitations. Please see Lines 450-455.

8. Comment: The manuscript would benefit from one final careful language edit to improve consistency in terminology and remove a few minor typographical and formatting inconsistencies.

Response: Many thanks for this comment. The manuscript was proofread and the language was edited. Please see Lines 341-455.

9. Comment: Overall, the manuscript has improved considerably since the previous version. After addressing the points above, I believe it will be suitable for publication.

Response: We sincerely thank you for your time, effort, and constructive feedback. We are very pleased to learn that the manuscript has improved considerably and is now you will recommend it for publication. All remaining points have been carefully addressed, and we are grateful for the guidance that has helped us refine the work to this stage.

Reviewer #2:

1. Comment: The author has appropriately addressed my questions, and I believe the submitted paper has been suitably revised. Good job.

Response: We sincerely thank the reviewer for the time and effort devoted to evaluating our revised manuscript. We are delighted that the reviewer has approved all revisions and recommended the acceptance of the revised manuscript.

Once again, we would like to thank you and the reviewers for the constructive feedback and comments, which we believe have improved the quality, clarity and flow of the revised manuscript.

Having addressed all comments and revised the manuscript accordingly, we are looking forward to hearing from you soon.

Kind regards

Ramzi Shawahna

Attachment Submitted filename: 03. Response to Reviewers.docx

# Editor report: Decision Letter 2

**DOI:** 10.1371/journal.pone.0358566.r005

**Authors:** Pawan Acharya

2 Sep 2026

Firearm injuries in a conflict affected region: A multicenter study of epidemiological trends, injury patterns, management, and outcomes in the West Bank of Palestine

PONE-D-26-16621R2

Dear Dr. shawahna,

We’re pleased to inform you that your manuscript has been judged scientifically suitable for publication and will be formally accepted for publication once it meets all outstanding technical requirements.

Within one week, you’ll receive an e-mail detailing the required amendments. When these have been addressed, you’ll receive a formal acceptance letter and your manuscript will be scheduled for publication.

An invoice will be generated when your article is formally accepted. Please note, if your institution has a publishing partnership with PLOS and your article meets the relevant criteria, all or part of your publication costs will be covered. Please make sure your user information is up-to-date by logging into Editorial Manager at Editorial Manager® and clicking the ‘Update My Information' link at the top of the page. For questions related to billing, please contact billing support.

If your institution or institutions have a press office, please notify them about your upcoming paper to help maximize its impact. If they’ll be preparing press materials, please inform our press team as soon as possible -- no later than 48 hours after receiving the formal acceptance. Your manuscript will remain under strict press embargo until 2 pm Eastern Time on the date of publication. For more information, please contact onepress@plos.org.

Kind regards,

Pawan Acharya, PhD, MSPH

Academic Editor

PLOS One

Additional Editor Comments (optional):

Reviewers' comments:

# Editor report: Acceptance letter

**DOI:** 10.1371/journal.pone.0358566.r006

**Authors:** Pawan Acharya

PONE-D-26-16621R2

PLOS One

Dear Dr. shawahna,

I'm pleased to inform you that your manuscript has been deemed suitable for publication in PLOS One. Congratulations! Your manuscript is now being handed over to our production team.

At this stage, our production department will prepare your paper for publication. This includes ensuring the following:

* All references, tables, and figures are properly cited

* All relevant supporting information is included in the manuscript submission,

* There are no issues that prevent the paper from being properly typeset

You will receive further instructions from the production team, including instructions on how to review your proof when it is ready. Please keep in mind that we are working through a large volume of accepted articles, so please give us a few days to review your paper and let you know the next and final steps.

Lastly, if your institution or institutions have a press office, please let them know about your upcoming paper now to help maximize its impact. If they'll be preparing press materials, please inform our press team within the next 48 hours. Your manuscript will remain under strict press embargo until 2 pm Eastern Time on the date of publication. For more information, please contact onepress@plos.org.

You will receive an invoice from PLOS for your publication fee after your manuscript has reached the completed accept phase. If you receive an email requesting payment before acceptance or for any other service, this may be a phishing scheme. Learn how to identify phishing emails and protect your accounts at https://explore.plos.org/phishing.

If we can help with anything else, please email us at customercare@plos.org.

Thank you for submitting your work to PLOS One and supporting open access.

Kind regards,

PLOS One Editorial Office Staff

on behalf of

Dr. Pawan Acharya

Academic Editor

PLOS One
